# Long Noncoding RNA *SNHG1* Regulates *LMNB2* Expression by Sponging *miR-326* and Promotes Cancer Growth in Hepatocellular Carcinoma

**DOI:** 10.3389/fonc.2021.784067

**Published:** 2021-11-30

**Authors:** Wentao Mu, Lingyu Guo, Yang Liu, Hui Yang, Shanglei Ning, Guoyue Lv

**Affiliations:** ^1^ Department of Hepatobiliary and Pancreatic Surgery, The First Hospital of Jilin University, Changchun, China; ^2^ Department of Hepatobiliary Surgery, General Surgery, Qilu Hospital of Shandong University, Jinan, China; ^3^ Department of Urology, Second Affiliated Hospital of Xi’an Jiaotong University, Xi'an, China; ^4^ Department of Hepatobiliary Surgery, Taian City Central Hospital of Shandong Province, Tai'an, China; ^5^ Department of Colorectal and Anal Surgery, The First Affiliated Hospital of Shandong First Medical University, Jinan, China

**Keywords:** hepatocellular carcinoma, *SNHG1*, *miR-326*, *LMNB2*, lncRNA, microRNA, bioinformatics

## Abstract

**Objective:**

The purpose of the study is to explore the potential competing endogenous RNA (ceRNA) network and investigate the molecular mechanism of long noncoding RNA (lncRNA) *small nucleolar RNA host gene 1* (*SNHG1*) in hepatocellular carcinoma (HCC) development.

**Methods:**

By analyzing the data of HCC in The Cancer Genome Atlas (TCGA) database, we included differentially expressed lncRNA and microRNA (miRNA) profiles and constructed ceRNA networks related to the prognosis of HCC patients. qRT-PCR, Western blotting, 3-(4,5-Dimethylthiazol-2-yl)-2,5-diphenyltetrazolium bromide (MTT), transwell assay, and the nude mouse model were employed to test the effects of *SNHG1* and *LMNB2* on tumor proliferation and growth *in vitro* and *in vivo*.

**Results:**

In the study, we identified 115 messenger RNAs (mRNAs), 12 lncRNAs, and 37 miRNAs by intersecting differentially expressed genes (DEGs) in TCGA and StarBase databases. Then, *SNHG1*–*miR-326*–*LMNB2* pathway came into notice after further survival analysis and hub gene screening. Our results showed that *SNHG1* expression was upregulated significantly in HCC tissues and cell lines. Downregulation of both *LMNB2*, the target of *miR-326* in HCC, and *SNHG1* inhibited tumor proliferation and growth *in vitro* and *in vivo*. Furthermore, *SNHG1* could regulate *LMNB2* expression through binding to *miR-326* in HCC cell lines.

**Conclusion:**

*SNHG1* is a promising prognostic factor in HCC, and the *SNHG1*–*miR-326*–*LMNB2* axis may be a potential therapeutic target for HCC.

## Introduction

Hepatocellular carcinoma (HCC) is the fourth most common cause of cancer-related death worldwide and the sixth leading cause of incident cancer cases (1, 2). The majority of HCC mainly occurs in patients with hepatitis B virus (HBV) infection, hepatitis C virus (HCV) infection, and alcohol abuse (3). Although the 5-year survival rate of early-stage HCC is about 60% these years, the prognosis of most patients with unresectable HCC is still very poor (4), despite recent advances in surgery and systematic therapy. Besides, HCC is a highly heterogeneous tumor that may be the main cause of treatment failure, so the biological diversity of HCC poses a considerable challenge for individualized therapy (5, 6). Therefore, it is urgent to explore the molecular mechanism of tumor progression and identify novel therapeutic targets for HCC.

Long noncoding RNAs (lncRNAs) are a set of RNA transcripts longer than 200 nucleotides that are not for translation but capable of regulating the expression of different genes (7). Emerging evidence has suggested that lncRNAs participate in tumor proliferation, invasion, apoptosis, and other biological processes (8–11). Generally, lncRNAs acted as a competing endogenous RNA (ceRNA) and bound with specific microRNA (miRNA), regulating the corresponding downstream messenger RNA (mRNA) translation (12–14). Although *SNHG1* has been reported to function as an oncogene through sponging *miR-195-5p* (15), *miR-377-3p* (16), and *miR-195* (17) in HCC, in the present study, we made use of The Cancer Genome Atlas (TCGA) database and identified a novel molecular mechanism of *SNHG1* in HCC growth.

Our data demonstrated the crucial roles of *SNHG1* in HCC proliferation and invasion *in vitro* and *in vivo*. Furthermore, we innovatively demonstrated that *SNHG1* promoted HCC growth through competitively binding to *microRNA-326* (*miR-326*) to regulate *LMNB2* expression, which provided a novel insight into the mechanism of HCC progression.

## Materials and Methods

### Data Source

The published HCC cohort dataset, including gene expression profiles and relevant clinical information, can be downloaded from TCGA data portal (18). The clinical information of patients includes age, gender, preoperative diagnosis, Child–Pugh score of liver function, radical resection, postoperative pathological diagnosis, pathological grading and staging, survival time, last follow-up time, and so on. Only the survival time-related data in the complex clinical data are used, so only the data needed in the article are shown in (**Supplementary Tables 1** and **2**). Data acquisition and application were conducted in accordance with TCGA release guidelines and data access policy without additional approval from the local ethics committee.

### Screening of Differentially Expressed mRNA, lncRNA, and miRNA

The limma package in R language (version 3.6.1) was subsequently used for the calculation of DEGs. Gene counts >0, the adjusted p value <0.05, and |log_2_ fold change|>1 were set as the cutoff criteria. Similarly, Differentially Expressed microRNAs (DEmiRs) were selected by the three cutoff values, Gene Counts >0, the adjusted p value <0.05, and |log_2_ fold change|>1. Heat maps and volcanic maps were drawn using R language. Then, the principal component analysis (PCA) figure about the samples was performed.

### Function and Pathway Enrichment Analysis

In order to understand the biological functions of selected DEGs and DEmiRs, we performed the enrichment analysis of DEGs and DEmiRs in Gene Ontology (GO) terms and Kyoto Encyclopedia of Genes and Genomes (KEGG) Database Pathways. The clusterProfiler (version 3.12.0) (19) package in R language was used for revealing the roles of DEGs in biological process (BP), cellular component (CC), and molecular function (MF). The adjusted p value was less than 0.01 and considered statistically significant.

### The ceRNA Network Construction

R language GDCRNATools package was used to search and match on StarBase database and find out the common miRNAs targeting mRNA and lncRNA (20, 21). The mRNA and lncRNA are negatively correlated with miRNA, but mRNA and lncRNA present a positive correlation. Hypergeometric distribution test was used to assess the importance between each pair of them. We used false discovery rate (FDR) to correct p values while FDR <0.05 is the cutoff value. Meanwhile, the regulation of similarity correlation with miRNAs (similarity of correlation between miRNA and lncRNA expression and correlation between miRNA and mRNA expression) is not equal to zero. All the competitive lncRNA and mRNA were mixed together after we identified them under the conditions above. Finally, ceRNA network diagram was established in the Cytoscape (3.7.2) for visualization.

### Establishment of ceRNA Network Associated With Hepatocellular Carcinoma Patient Survival

In order to find out the survival-related lncRNA–miRNA–mRNA pathways, we conducted a single-factor survival analysis on each lncRNA, mRNA, and miRNA node in the ceRNA network. The node was marked in the ceRNA network diagram, and the survival-related ceRNA network was constructed through the survival-related node. Then, samples were divided into the high-expression group and the low-expression group according to the median expression of each gene. The Kaplan–Meier survival was used to evaluate the difference of overall survival (OS) time between the two groups. Finally the *SNHG1*–*miR-326*–*LMNB2* pathway was selected, and the effects of this pathway were experimentally verified in our study.

### Cell Lines

The human hepatocellular cancer cell lines (Huh7 and PLC) were obtained from the Cell Bank of Chinese Academy of Sciences, Shanghai Branch. The Huh7 and PLC cells were cultured in Dulbecco’s modified Eagle’s medium (DMEM) with 10% fetal bovine serum in 5% CO_2_ and 90% humidity at 37°C.

### Cell Transduction

The lentivirus vectors (sh-*SNHG1*, sh-*LMNB2*) and small interfering RNAs (siRNAs) against human *SNHG1* or *LMNB2* were synthesized by GenePharma Co. Ltd. The stable Huh7 cells with *SNHG1* and *LMNB2* knocked down were generated using lentiviral vectors. Infected cells were then treated with puromycin (2 µg/ml) for 2 days, and surviving cells were maintained in complete medium with puromycin (0.5 µg/ml). The siRNAs were transfected into hepatocellular cancer cells using Lipofectamine 2000 (Thermo Fisher Scientific, Inc.) according to their instructions. Besides, *miR-326* mimics, *miR-326* inhibitors, and negative controls were purchased from GenePharma Co. Ltd. When the cell confluence reached 50%, oligonucleotide transfection was performed using Lipofectamine 2000 according to the manufacturer’s protocol.

### Luciferase Reporter Assay

We constructed the wild-type plasmid *SNHG1*-WT and the mutant plasmid *SNHG1*-MUT. PLC and HuH7 cells that were seeded in 24-well plates were cotransfected with *miR-326* mimic or negative control and wild-type or mutant plasmids using Lipofectamine 2000. The luciferase intensity on the microplate was measured with the Dual-Luciferase Reporter Assay System (Promega Corp.), and Renilla luciferase activity was normalized to firefly luciferase activity.

### Western Blotting

The total protein was extracted from the cells using radioimmunoprecipitation assay lysis buffer with protease inhibitors. Each lane is loaded with the same amount of total protein (20 μg), and the sample is separated by 10% sodium dodecyl sulfate polyacrylamide gel electropheresis (SDS PAGE) and then transferred to a polyvinylidene fluoride membrane (Roche Diagnostics). After blocking with 5% skim milk at room temperature for 1 h, primary antibody *LMNB2* (Abcam) or *glyceraldehyde 3-phosphate dehydrogenase* (*GAPDH*; Abcam) was used at 4°C overnight. Subsequently, the membranes were incubated with anti-rabbit (1:3,000, cat. no. 7074, Cell Signaling Technology, Inc.) horseradish peroxidase-conjugated secondary antibodies at 37°C for 1 h. Finally, ECL Western blot substrate (Promega corp.) and FluorChem E system (Protein Simple) were used to observe the immune response zone.

### qPCR

According to the manufacturer’s protocol, TRIzol reagent (Thermo Fisher Scientific, Inc.) was used to extract total RNA from cells and tissues. The RNA purity was evaluated based on the A260/280 ratio. RNA was reverse transcribed into cDNA using miRNA first-strand cDNA synthesis kit [Accurate Biotechnology (Hunan) Co., Ltd.]. SYBR Green Premix Pro Taq HS qPCR Kit II [Accurate Biotechnology (Hunan) Co., Ltd.] was used for qPCR to detect the relative expression of the target gene. Thermal cycling conditions are as follows: Initial denaturation at 95°C for 30 sec; 40 cycles of 5 sec at 95°C; 1 min at 60°C and 72°C for 15 sec; with a final extension cycle at 72°C for 5 min. Finally, it step in the dissociation stage. The relative levels were calculated using the 2^-ΔΔCq^ method. The endogenous control gene is GAPDH. The primer sequence is shown in **Table 1**.

**Table 1:** qPCR primers.

	Forward Primer	Reverse Primer
miR-326	CATCTGTCTGTTGGGCTGGA	AGGAAGGGCCCAGAGGCG
SNHG1	CTACTGACCTAGCTTGTTGCCA	GGCCCTGAATGAGCTACCTAC
U6	CTCGCTTCGGCAGCACA	AACGCTTCACGAATTTGCGT
GAPDH	GCACCGTCAAGGCTGAGAAC	TGGTGAAGACGCCAGTGGA
LMNB2	CTGGAGCTGGAGCAGACCTA	TCCCGAATGCGATCTTCAGC

### Cell Migration and Invasion Assays

Cells (1 × 10^5^) were seeded in the upper chamber of a Boyden chamber (8 μm aperture) in 200 μl of serum-free medium (Corning Corporation). The lower chamber was filled with 700 μl containing 10% fetal bovine serum (Gibco) as a chemical attractant. After 24 h of incubation at 37°C, cells remaining on the upper side of the membrane were removed with a cotton swab, and the cells that had migrated to the lower side of the membrane were fixed with 70% ethanol for 20 min and stained with 0.1% crystal violet for 20 min at room temperature. Then, the cells were counted with an optical microscope. The method of the invasion test is similar to cell migration assay except that the Boyden chamber is covered with a matrix before seeding the cells.

### Wound Healing Test

The cells were seeded in six-well plates and cultured to 80% confluence before we scratched the cell layer with a 20-μl pipette tip. The cells were then incubated in fresh medium containing 10% fetal bovine serum for 48 h. Scratches were observed under a fluorescence microscope after 24 and 48 h.

### *In Vivo* Assay

Female nude mice (aged 5–6 weeks, weight 18–22 g) were purchased from Hangzhou Ziyuan Biology and bred in a 12-h light/dark cycle and sterile conditions (temperature 26°C–28°C, humidity 40%–60%) with free access to water and food. We injected 3 × 10^6^ cells into the fore limbs of nude mice to generate transplanted tumors and measure the tumor size with a caliper every 3 days. After 19 days, the mice were sacrificed, and tumor images were captured. Tumor volume was calculated with the formula: maximum diameter × (minimum diameter) 2 × 0.5.

### Statistical Analysis

Statistical analysis was performed using SPSS 19.0 software (SPSS Inc., Chicago, IL, USA). The summary data are expressed as the mean ± standard error of the mean (SEM). We use the χ^2^ test, Student’s t-test, or one-way analysis of variance with the least significant difference correction to assess the differences between groups. Spearman rank correlation analysis was used to evaluate linear regression. p value less than 0.05 was considered to be statistically significant in all cases.

### Ethics Approval and Consent to Participate

The study protocol was approved by the research ethics committee of the Qilu Hospital of Shandong University.

## Results

### Identification of Differentially Expressed Genes and Differentially Expressed microRNAs (DEmiR)

**Figure 1:**
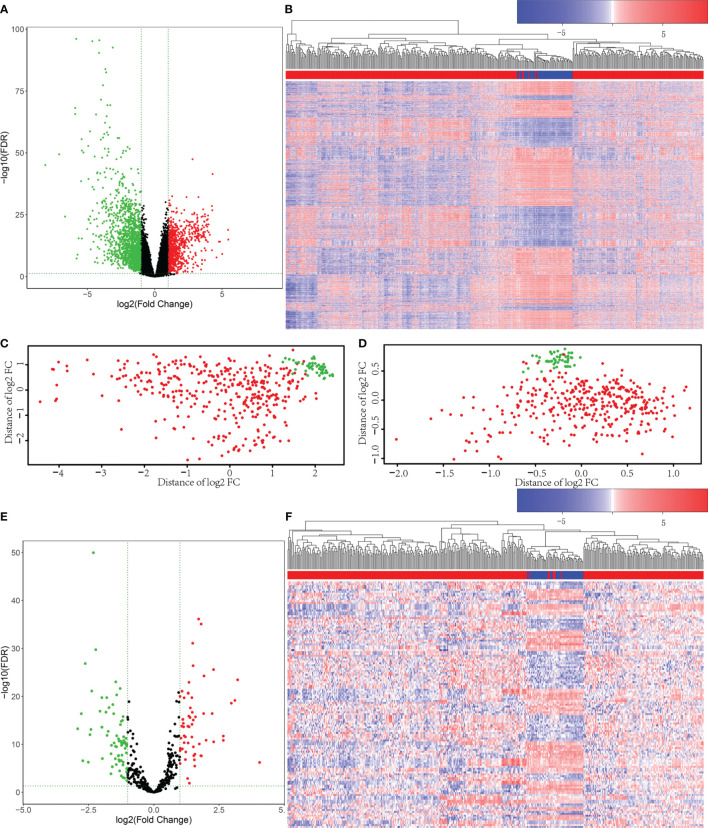
Differentially expressed mRNAs and miRNAs were selected from The Cancer Genome Atlas (TCGA) database. **(A)** Volcano plots for 2,416 differentially expressed genes (DEGs). The green dots represent downregulated genes, and the red dots represent upregulated genes. Black dots represent genes without statistically significant change (p < 0.05 and |log2 fold change|>1). **(B)** Heatmap for DEGs in primary tumor (red) compared with solid tissue normal (blue). **(C)** The principal component analysis (PCA) figure of the samples based on the differentially expressed mRNAs. **(D)** The PCA figure of the samples based on the differentially expressed miRNAs. **(E)** Volcano plots for the 131 Differentially Expressed microRNAs (DEmiRs). The green dots represent downregulated miRNAs, and the red dots represent upregulated miRNAs. Black dots represent miRNAs without statistically significant change (p < 0.05 and |log2 fold change|>1). **(F)** Heatmap for DEmiRs in primary tumor (red) compared with solid tissue normal (blue).

Firstly, 421 HCC patient samples with para-tumor tissue available from TCGA database were analyzed using R language tools. We used p value <0.05 and |log_2_ fold change|>1 as cutoffs to identify differential gene profiles. As a result, a total of 2,416 differentially expressed genes (DEGs), 2,181 differentially expressed mRNAs, and 148 differentially expressed lncRNAs were identified (**Figures 1A–C**). The green dots represent downregulated genes, and the red dots represent upregulated genes. Black dots represent genes without statistically significant change. The heatmap shows the DEGs between the primary tumor (red) and solid tissue normal (blue). Moreover, 131 differentially expressed miRNAs were screened using both R language and GDCRNATools (**Figures 1D–F**).

### Gene Ontology Enrichment Analysis and Kyoto Encyclopedia of Genes and Genomes Enrichment Analysis

**Figure 2:**
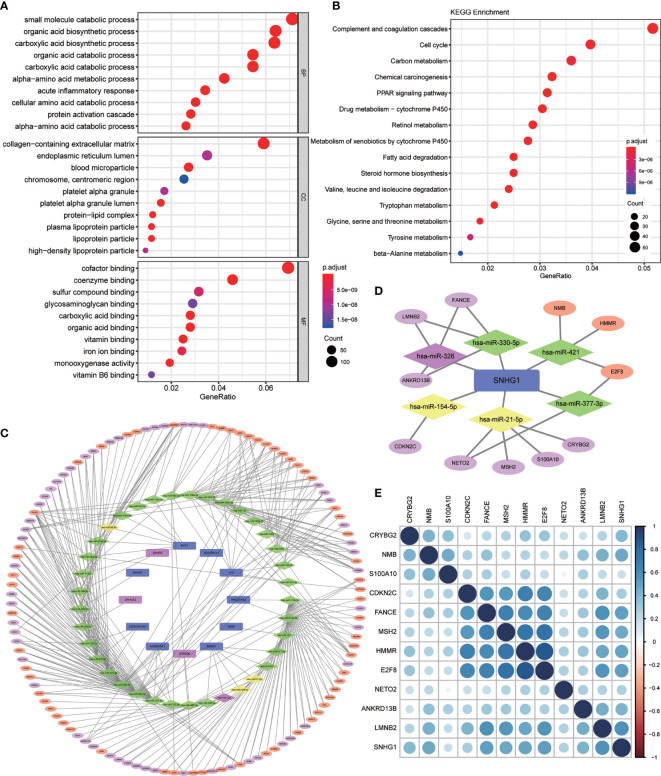
Differential gene enrichment analysis and pathway construction. **(A)** Gene Ontology (GO) enrichment analysis of differentially expressed genes (BP, biological process; CC, cellular component; MF, molecular function) (p < 0.01). **(B)** Kyoto Encyclopedia of Genes and Genomes (KEGG) pathway analysis of differentially expressed genes (p < 0.01). **(C)** Competing endogenous RNA (ceRNA) network was established by Cytoscape for differentially expressed lncRNA–miRNA–mRNA. (All lncRNA nodes and mRNA nodes were differentially expressed; purple nodes were associated with survival. Yellow miRNA nodes were differentially expressed.). **(D)** A total of six miRNAs and 11 mRNAs were identified from the lncRNA *SNHG1*-related network. **(E)** Heatmap for correlation matrix to visualize the gene expression correlation quantitatively.

GO and KEGG enrichment analysis on these 2,416 DEGs was carried out to explore their functions (p value <0.01 was the cutoff value). The results showed that (**Figure 2A**) the differential genes mainly focus on the catabolic process of biological procedure extracellular matrix of cells and small molecular compounds bindings with the oxidative respiratory chains. Otherwise, KEGG enrichment analysis demonstrated that the differential genes concentrated upon the pathways participating in complement and coagulation cascades, cell cycle regulation, and cell metabolism (**Figure 2B**).

### Construction of the ceRNA Network

Based on the 2,181 differentially expressed mRNAs and 148 differentially expressed lncRNAs mentioned above, we constructed the ceRNA network containing 115 mRNAs, 12 lncRNAs, and 37 miRNAs, in which 343 different lncRNA–miRNA–mRNA pathways were also described (**Figure 2C**). Then, we intersected the selected 37 miRNAs with DEmiRs obtained previously and only four differentially expressed miRNAs including *miR-326*, *miR-154-5p*, *miR-21-5p*, and *miR-93-5p* were found, which means that these four miRNA-associated lncRNA–miRNA–mRNA pathways may function in HCC development.

### Survival Analysis of Candidate lncRNA–miRNA–mRNA Pathway in Hepatocellular Carcinoma Patients

**Figure 3:**
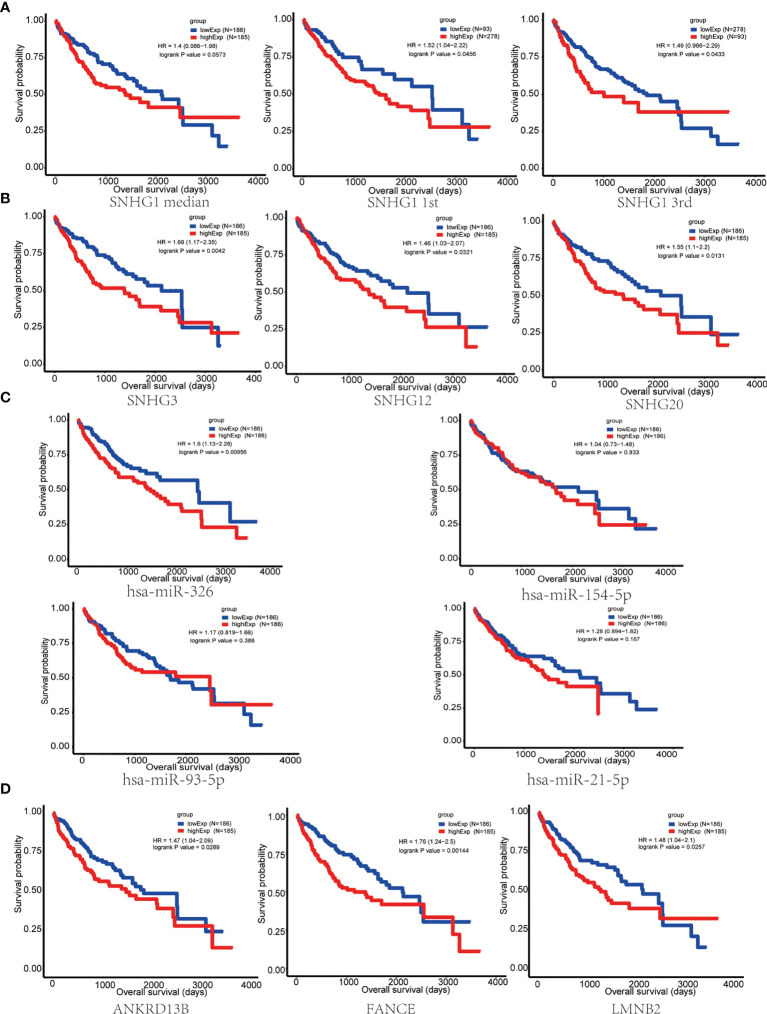
Survival analysis of key nodes in hepatocellular carcinoma (HCC). **(A)** Kaplan–Meier curve of HCC patients with low or high *SNHG1* expression of the quartile subgroups. **(B)** *SNHG3*, *SNHG1*2, and *SNHG20* were significantly associated with the overall survival in patients with HCC, as detected using the Kaplan–Meier curve. **(C)** Kaplan–Meier curve of HCC patients with low or high expression of *miR-326*, *miR-154-5p*, *miR-21-5p*, and *miR-93-5p*. **(D)** The Kaplan–Meier curve showed that *ANKRD13B*, *FANCE*, and *LMNB2* were significantly associated with the overall survival in patients with HCC.

**Figure 4:**
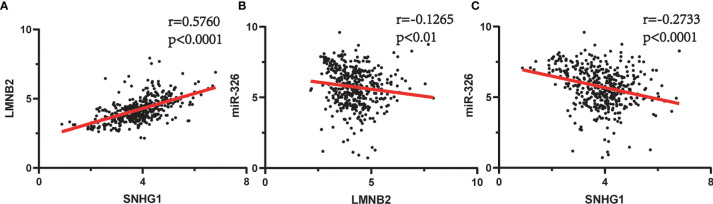
Spearman’s correlation analysis between *SNHG1*, *miR-326*, and *LMNB2* in hepatocellular carcinoma (HCC). **(A)** *SNHG1* is positively correlated with *LMNB2* (r = 0.5760, p < 0.0001). **(B)** *miR-326* is negatively correlated with *LMNB2* (r = 0.1256, p < 0.01). **(C)** *SNHG1* is negatively correlated with *miR-326* (r = 0.2733, p < 0.0001).

In order to evaluate the prognostic significance of the obtained ceRNA network, we used univariate survival analysis, Cox regression, and Kaplan–Meier survival analysis and found that only *miR-326* was associated with HCC patient survival among the four potential miRNAs (p < 0.01) (**Figure 3C**). So, the lncRNA–miRNA–mRNA pathways that associated with *miR-326* were extracted from ceRNA as a whole (**Figure 2D**). And the heatmap of correlation matrix was drawn to visualize the gene expression correlation quantitatively (**Figure 2E**). We evaluated the prognostic significance of SNHG family members, such as *SNHG1 SNHG3*, *SNHG1*2, and *SNHG20*; although the p value for *SNHG1* survival analysis is slightly greater than 0.05, the survival analysis p values for 25% and 75% of the quartile subgroups were both less than 0.05 (**Figures 3A, B** **)**. Furthermore, previous studies have demonstrated that lncRNA *SNHG1* predicted a poor prognosis in HCC (16, 22), and the diagram showed that lncRNA *SNHG1* is a potential lncRNA in HCC progression. Although three candidate targets *ANKRD13B*, *LMNB2*, and *FANCE*, which were regulated by *miR-326*, were all associated with HCC patient survival (**Figure 3D**), only *LMNB2* has been investigated in a hepatocellular cancer study before (23). Previous studies showed that *ANKRD13B* and *FANCE* were associated with epidermal growth factor receptor (EGFR) activation (24) and Fanconi anemia (25, 26), respectively. Furthermore, correlation analysis indicated both lncRNA *SNHG1* and *LMNB2* were negatively correlated with *miR-326*, but *SNHG1* correlated with *LMNB2* positively in HCC (**Figures 4A–C**).

### *SNHG1* Promotes the Proliferation, Migration, and Invasion of Hepatocellular Cancer Cells

**Figure 5:**
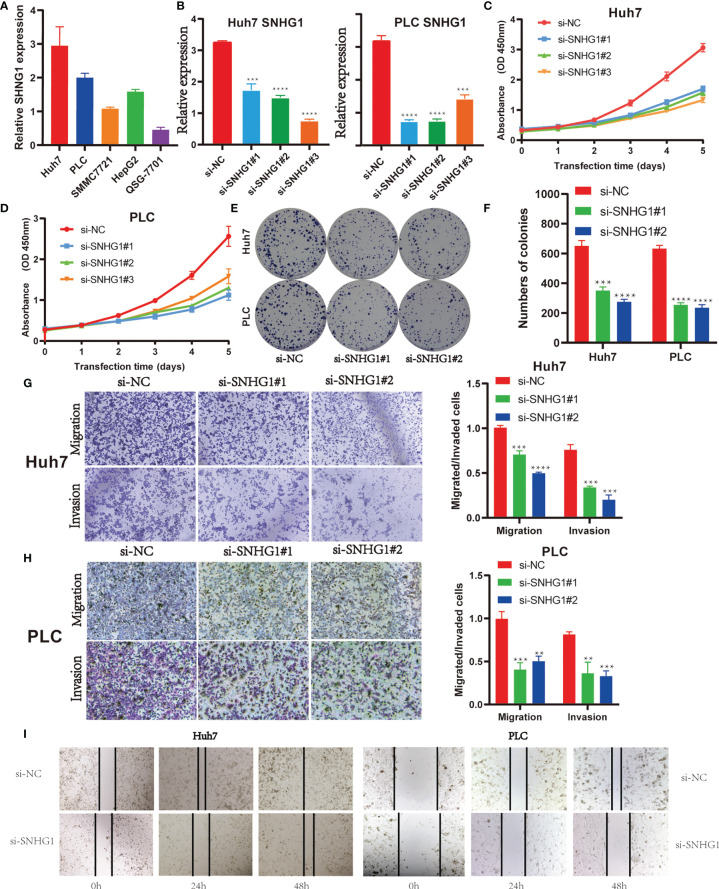
*SNHG1* promotes proliferation, migration, and invasion of hepatocellular cancer cells. **(A)** Relative expression of *SNHG1* was detected in QSG-7701 and the other four hepatocellular carcinoma (HCC) cell lines by RT-qPCR. **(B)** Expression of *SNHG1* in Huh7 and PLC cells with *SNHG1* knockdown detected by RT-qPCR (***p < 0.001, ****p < 0.0001). **(C)** Optical density (OD) value of Huh7 cells with *SNHG1* knockdown and control cells in 3-(4,5-Dimethylthiazol-2-yl)-2,5-diphenyltetrazolium bromide (MTT) assay. **(D)** OD value of PLC cells with *SNHG1* knockdown and control cells in MTT assay. **(E)** Colonies of Huh7 and PLC cells with *SNHG1* knockdown and control cells in the colony formation assay. **(F)** The number of colonies was calculated 14 days after cell seeding (***p < 0.001, ****p < 0.0001). **(G)** Migratory and invasive potential of Huh7 cells with *SNHG1* knockdown and control cells (***p < 0.001, ****p < 0.0001). **(H)** Migratory and invasive potential of PLC cells with *SNHG1* knockdown and control cells (**p < 0.01, ***p < 0.001). **(I)** Wound healing assay was used to detect the migratory ability of Huh7 and PLC cells with *SNHG1* knockdown.

Firstly, *SNHG1* level were examined in five HCC cell lines. As shown in **Figure 5A**, Huh7 and PLC have a higher expression of *SNHG1*, so we chose the two cell lines for further research. Three independent siRNAs were designed to silence *SNHG1* expression. The results showed that *SNHG1* expression decreased significantly compared with control group (**Figure 5B**). MTT assay and colony formation demonstrated that *SNHG1* silencing inhibited cell proliferation significantly (**Figures 5C–F**). The transwell assay indicated that knockdown of *SNHG1* inhibited the migration and invasion of Huh7 and PLC cells (**Figures 5G, H** **)**, and these results were confirmed in the wound healing experiment using both Huh7 and PLC cells (**Figure 5I**). The above results showed that *SNHG1* promoted the proliferation, migration, and invasion of hepatocellular cancer cells.

### *SNHG1* Knockdown Inhibits Tumor Growth *In Vivo*

**Figure 6:**
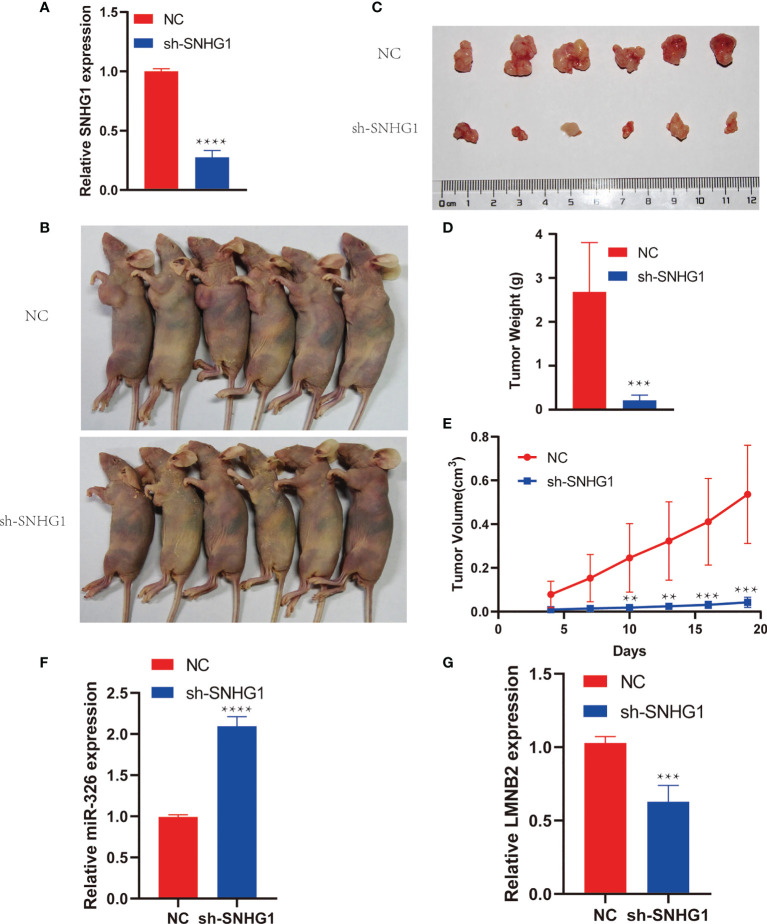
*SNHG1* promotes hepatocellular carcinoma (HCC) tumor growth *in vivo*. **(A)** RNA levels of *SNHG1* in Huh7 cells with *SNHG1* knockdown detected by RT-qPCR (****p < 0.0001). **(B)** Representative images of mice bearing tumors from *SNHG1* knockdown group and control group. **(C)** Representative tumor images were shown. **(D, E)** Quantification of tumor weight and tumor volume of individual mice following the injection of HuH7 cells with *SNHG1* knockdown or control cells (**p < 0.01, ***p < 0.001). **(F, G)** Expression of *miR-326* and *LMNB2* in tumor of sh*SNHG1* group compared with control (***p < 0.001, ****p < 0.0001).

To investigate the role of *SNHG1* in HCC growth *in vivo*, we cultured Huh7 cells stably expressing sh*SNHG1* using lentivirus (**Figure 6A**). As shown in **Figures 6B, C** (six mice per group), the knockdown of *SNHG1* inhibited tumor growth significantly compared with control group (**Figures 6A, B** **)**. Furthermore, decreased tumor size and weight were observed after *SNHG1* was knocked down (**Figures 6D, E** **)**. These results indicated that *SNHG1* knockdown could influence tumor growth significantly *in vivo*. The *miR-326* level of tumor increased markedly in the sh*SNHG1* group compared with control, whereas the expression of *LMNB2* decreased (**Figure 6F, G** **)**.

### SNHG1 Regulates LMNB2 Expression in Hepatocellular Carcinoma *via* Sponging *miR-326* in Hepatocellular Carcinoma

**Figure 7:**
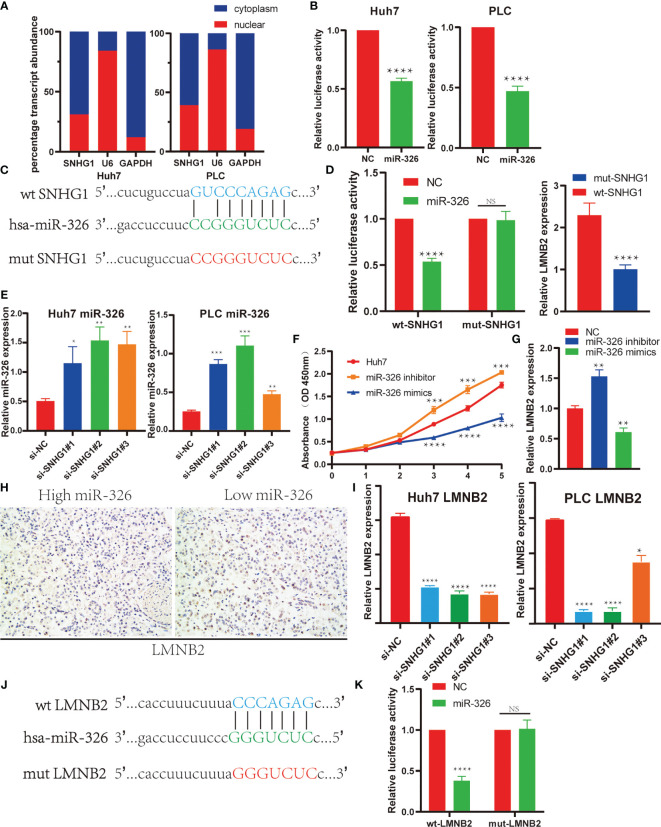
*SNHG1* regulated *LMNB2* expression through sponging *miR-326* in hepatocellular carcinoma (HCC) cells. **(A)** Subcellular localization of *SNHG1* was detected by quantifying nuclear and cytoplasmic fractions. U6 and glyceraldehyde 3-phosphate dehydrogenase (GAPDH) were used as nuclear and cytosolic controls, respectively. **(B)** Dual-luciferase reporter assay showed that *miR-326* directly interacted with *SNHG1* in Huh7 and PLC cells (****p < 0.0001). **(C)** The potential binding site of *SNHG1* and *miR-326* predicted by StarBase. The sequence alignment of *miR-326* and the predicted binding region in *SNHG1* (green). Predicted *miR-326* binding sites (blue) in *SNHG1* and position of mutated nucleotides in *SNHG1* (red). **(D)** Dual-luciferase reporter assay was performed to detect the binding of *miR-326* with WT-*SNHG1* in Huh7 cells. Expression of *LMNB2* in Huh7 cells after addition of wt-*SNHG1* compared with mut-*SNHG1* (****p < 0.0001). **(E)** Expression of *miR-326* in Huh7 and PLC cells with *SNHG1* knockdown (*p < 0.05, **p < 0.01, ***p < 0.001). **(F)** OD value of Huh7 cells in the MTT assay demonstrated that *miR-326* mimics could inhibit cell proliferation significantly, while *miR-326* inhibitor could accelerate HuH7 cell proliferation (***p < 0.001, ****p < 0.0001). **(G)** Expression of *LMNB2* in Huh7 cells transfected with *miR-326* mimics and inhibitor was measured by qRT-PCR (**p < 0.01). **(H)** Representative immunohistochemistry (IHC) images of *LMNB2* are shown in hepatocellular carcinoma with different *miR-326* expression levels. **(I)** RNA levels of *LMNB2* in Huh7 and PLC cells with *SNHG1* knockdown detected by RT-qPCR (*p < 0.05, ****p < 0.0001). **(J)** The potential binding site of *LMNB2* and *miR-326* predicted by StarBase. The sequence alignment of *miR-326* and the predicted binding region in *LMNB2* (green). Predicted *miR-326* binding sites (blue) in *LMNB2* and position of mutated nucleotides in *SNHG1* (red). **(K)** Dual-luciferase reporter assay was performed to detect the binding of *miR-326* with wt-*LMNB2* in Huh7 cells (****p < 0.0001). NS, No Significant.

In order to confirm the regulatory axis of *SNHG1*–*miR-326*–*LMNB2* in HCC mentioned above, we firstly perform the subcellular localization assay of *SNHG1*. Nucleocytoplasmic separation and RT-qPCR assay revealed that *SNHG1* was expressed in both nucleus and cytoplasm of HuH7 and PLC cells, whereas a larger proportion of *SNHG1* was located in the cytoplasm (**Figure 7A**). Many lncRNAs acted as ceRNAs through binding miRNAs (27). *SNHG1* has also been reported to be involved in tumor development including colorectal cancer and osteosarcoma (28–30). Therefore, we used dual-luciferase reporter assays to make sure whether *SNHG1* can directly interact with *miR-326* in Huh7 and PLC cells. The results showed that the *miR-326* mimics could reduce the luciferase activity in both Huh7 and PLC cells (**Figure 7B**). Meanwhile, wild-type and mutant *SNHG1* luciferase reporter vectors were constructed using binding sites predicted by StarBase (**Figure 7C**), and it was found that *miR-326* mimics reduced the luciferase activity significantly of wt-*SNHG1* but showed no effect on mut-*SNHG1*. Furthermore, addition of wt-*SNHG1* will attenuate the inhibition effect of *miR326* on the *LMNB2* expression level, while addition of mut-*SNHG1* will not (**Figure 7D**). Actually, *miR-326* level increased markedly after *SNHG1* was knocked down in HuH7 and PLC cells (**Figure 7E**). These results were consistent with the correlation described in **Figure 4**. Additionally, *miR-326* mimics could inhibit cell proliferation significantly, while *miR-326* inhibitor could accelerate HuH7 cell proliferation (**Figure 7F**). Bioinformatics analysis has revealed that *LMNB2* is the potential target of *miR-326* in HCC. To test the effect of *miR-326* on *LMNB2* expression, as the results shown in **Figure 7G**, *LMNB2* level decreased markedly with *miR-326* mimic treatment in Huh7 cells compared with control, whereas the expression of *LMNB2* increased significantly with *miR-326* inhibitor treatment. In a word, *miR-326* could downregulate *LMNB2* expression in HCC. Moreover, *LMNB2* was observed to be upregulated in the HCC tissues with low expression of *miR-326* (**Figure 7H**). Furthermore, the *LMNB2* expression was also investigated after *SNHG1* silence in HCC cells (**Figure 7I**). Moreover, wild-type and mutant *LMNB2* luciferase reporter vectors were constructed using binding sites predicted by StarBase (**Figures 7J, K** **)**, and it was found that *miR-326* mimics significantly reduced the luciferase activity of wt-*LMNB2* but showed no effect on mut-*LMNB2*. The data showed that *LMNB2* RNA expression was reduced significantly after *SNHG1* siRNA treatment. To sum up, *LMNB2* is the target of *miR-326* in HCC, and *SNHG1* regulates *LMNB2* expression *via* sponging *miR-326* in HCC.

### *LMNB2* Mediated the Positive Effects of *SNHG1* on Tumor Growth in Hepatocellular Carcinoma

**Figure 8:**
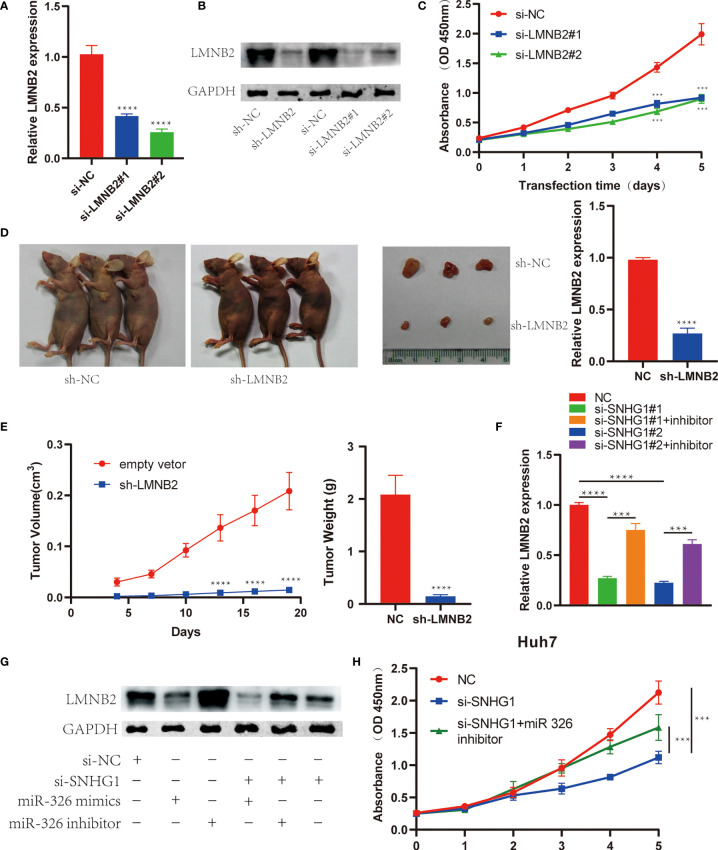
*LMNB2* promotes hepatocellular carcinoma (HCC) growth and mediates the role of *SNHG1* in HCC. **(A)** *LMNB2* expression decreased significantly in Huh7 cells with *LMNB2* siRNA treatment (****p < 0.0001). **(B)** Western blotting was performed to detect the protein level of *LMNB2* in Huh7 cells. **(C)** OD value of Huh7 cells with *LMNB2* knockdown and control cells in the 3-(4,5-Dimethylthiazol-2-yl)-2,5-diphenyltetrazolium bromide (MTT) assay (***p < 0.0001). **(D, E)** Quantification of tumor volume and tumor weight of mice following the injection of HuH7 cells with *LMNB2* knockdown or control cells. Representative images of mice bearing tumors, and tumor images were shown (****p < 0.0001). **(F)** The qRT-PCR demonstrated that *miR-326* inhibitor rescued the decreased *LMNB2* expression caused by *SNHG1* knockdown (***p < 0.001, ****p < 0.0001). **(G)** Western blotting assay showed that *miR-326* regulated *LMNB2* expression, and *miR-326* inhibitor rescued *LMNB2* downregulation caused by *SNHG1* knockdown in Huh7 cells. **(H)** MTT assays demonstrated that *miR-326* inhibitor rescued tumor growth inhibition caused by *SNHG1* knockdown (***p < 0.0001).

In order to explore the biological effects of *LMNB2* in HCC, we also knocked down *LMNB2* expression using siRNAs or lentivirus-mediated shRNA. The results showed that *LMNB2* expression was reduced in HuH7 cells significantly from RNA to protein level (**Figures 8A, B** **)**. It is worth noting that the proliferation ability of Huh7 cells decreased markedly with the silence of *LMNB2* expression (**Figure 8C**). Furthermore, we further studied the role of *LMNB2* on tumor growth *in vivo* (three mice per group). Twenty days after transfected HuH7 cells were injected into nude mice, the volume and weight of formed tumor were significantly smaller than those of control group (**Figures 8D, E** **)**, also indicating that *LMNB2* knockdown could inhibit the growth of HCC cells *in vitro* and *in vivo*. Besides, *SNHG1* knockdown reduced *LMNB2* expression compared with control in Huh7 cells, but *SNHG1*-induced downregulation of *LMNB2* was reversed or enhanced by *miR-326* inhibitor or mimic treatment, respectively. The similar results were also confirmed by Western blotting assays (**Figures 8F, G** **)**. In addition, MTT assay proved that *miR-326* inhibitor could rescue the role of *SNHG1* knockdown on HuH7 cell proliferation. (**Figure 8H**). These data demonstrated that the oncogenic *SNHG1* upregulated *LMNB2* expression and promoted tumor growth *via* suppressing *miR-326* level in HCC.

## Discussion

The present study was conducted to investigate potential lncRNA–miRNA–mRNA regulatory network based on TCGA database and elucidate the molecular signatures of HCC progression. Firstly, we screened 2,416 DEGs from RNA-seq of TCGA database through R language, including 148 lncRNAs and 2,181 mRNAs. After analyzing the miRNA data, a total of 131 differentially expressed miRNAs were screened. Then, GO enrichment analysis of DEGs indicated that differential genes are mainly concentrated in the small molecule catabolic process, collagen-containing extracellular matrix, and small molecular compounds bindings in the oxidative respiratory chain of molecular function. The results from KEGG enrichment analysis of DEGs showed that the enrichment of differential genes focused on the pathways associated with complement and coagulation cascades, cell cycle, and carbon metabolism. The GDCRNA Tools package was used to search and match in StarBase database the R language to figure out the miRNAs that linked lncRNAs with mRNAs described above. Finally, 164 related nodes and 343 correlations were found that contained 12 lncRNAs, 115 mRNAs, and 37 miRNAs. We utilized Cytoscape to visualize the relationships between them through the relational network. Then, we intersected the 37 miRNAs selected by DEGs with DEmiRs obtained previously; as a result, only four differentially expressed miRNAs were left including *miR-326*, *miR-154-5*p, *miR-21-5p*, and *miR-93-5p*. Univariate survival analysis showed that only *miR-326* was associated with OS among the four differentially expressed miRNAs. So the lncRNA–miRNA–mRNA axis that contained *miR-326* was extracted from the network, and we could see the direct relationship between *SNHG1* and *miR-326* in HCC; at the same time, *LMNB2*, *FANCE*, and *ANKRD13B*, three candidate targets, were involved in the diagram. In view of the reported role of *LMNB2* in HCC progression, recently, we selected *SNHG1*–*miR-326*–*LMNB2* axis as the hypothetic signaling involved in HCC development and progression (23).

Emerging evidence showed that lncRNAs acted as oncogenes or tumor suppressors in different types of cancer through regulating gene expression (31). Recently, many studies have unveiled the crucial role of *SNHG1* in various cancer tumorigeneses and progressions (28, 30, 32). In the present study, we found that *SNHG1* expression level was higher in HCC tissues and cancer cell lines and was a poor prognosis marker in HCC, which is consistent with previous reports (33–35). Furthermore, knockdown of *SNHG1* expression could inhibit cell proliferation, migration, and invasion significantly, and the function of *SNHG1* was further confirmed in an *in vivo* xenograft model. However, the mechanism of *SNHG1* in HCC is still unclear. Zhang et al. (35) revealed that *SNHG1* promoted HCC proliferation and cell cycle progression through inhibiting *p53* and its target genes expression, and similar results were described in colorectal cancer (36). Recent further research found that binding to *DNMT1* mediated the role of *SNHG1*-induced *p53* inhibition in HCC (34). As we know, sponging miRNA is an important regulatory way of lncRNA functions. For example, *SNHG1* has been reported to regulate *PDCD4* expression by sponging *miR-195-5p* in HCC (15). Recent studies found that *SNHG1* promoted HCC progression *via* sponging *miR-377-3p* and *miR-195* (16, 17). Interestingly, our findings showed a new mechanism through which *SNHG1* promoted HCC growth by binding to *miR-326* directly. Our data indicated that *miR-326* level increased significantly after *SNHG1* knockdown and *SNHG1* level is correlated with *miR-326* expression negatively based on TCGA database. Consistent with our reports, *SNHG1* also accelerated tumorigenesis by sponging *miR-326* in osteosarcoma and promoted nucleus pulposus cell proliferation through regulating *miR-326* (30, 37).

*Lamin B2* is a member of the lamin protein family known as the nuclear lamina, which included *lamin A*, *B1*, *B2*, *B3*, and *C* (38). It is reported to be involved in the formation of mitotic spindles (39). However, increasing evidence showed that *LMNB2* was associated with prostate and lung tumor progression and served as a prognostic marker (40–42). In our results, we also found that knockdown of *LMNB2* inhibited cell proliferation and growth *in vitro* and *in vivo*, consistent with the data in latest reports (23). miRNAs are small noncoding RNAs that regulate gene expression negatively by enhancing degradation of the target mRNA and inhibiting the following translation (43). Based on our data in bioinformatics analysis and cell experiments, we found that *miR-326* mimics could reduce *LMNB2* mRNA and protein expression compared with control in HCC cell lines, meaning we firstly identified *LMNB2* as the target of *miR-326* in HCC. To verify the hypothesis that *SNHG1* regulates *LMNB2* expression by sponging *miR-326* in HCC, we tested the effects of *miR-326* on *SNHG1*-induced *LMNB2* expression. The rescue experiments indicated that *miR-326* inhibitor reversed the *LMNB2* decrease induced by *SNHG1* knockdown, and correlation analysis also confirmed the *SNHG1*–*miR-326*–*LMNB2* axis in HCC. Although Zhang et al. (42) have demonstrated that *LMNB2* was responsible for the malignant phenotype of non-small cell lung carcinoma (NSCLC) through upregulating demethylation of *H3K9*, the mechanism of *SNHG1* in HCC progression still needs further investigation.

To sum up, we found that *SNHG1* acted as a ceRNA by sequestering miR-326 and regulating *LMNB2* expression in HCC. These findings contributed to a better understanding of the mechanisms underlying HCC progression. *SNHG1* may be a promising biomarker for predicting prognosis and a potential therapeutic target for HCC.

## Data Availability Statement

The datasets presented in this study can be found in online repositories. The names of the repository/repositories and accession number(s) can be found in the article/**Supplementary Material**.

## Ethics Statement

The studies involving human participants were reviewed and approved by Qilu Hospital of Shandong University. The patients/participants provided their written informed consent to participate in this study. The animal study was reviewed and approved by Qilu Hospital of Shandong University.

## Author Contributions

WM, LG, and SN participated in the conceptual design of the study. WM participated in the operation of bioinformatics tools. WM, LG, and SN performed the experiments. SN and HY are responsible for fund acquisition. GL is responsible for resource acquisition. LG, YL, and GL participated in the data verification. WM and SN performed the data analysis and wrote the article. All authors have read and reviewed the article. All authors contributed to the article and approved the submitted version.

## Funding

The present study was supported by the National Natural Science Foundation of China (grant nos. 31701013 and 81903044) and the Natural Science Foundation of Shandong Province (grant no. ZR2017BC032).

## Conflict of Interest

The authors declare that the research was conducted in the absence of any commercial or financial relationships that could be construed as a potential conflict of interest.

## Publisher’s Note

All claims expressed in this article are solely those of the authors and do not necessarily represent those of their affiliated organizations, or those of the publisher, the editors and the reviewers. Any product that may be evaluated in this article, or claim that may be made by its manufacturer, is not guaranteed or endorsed by the publisher.

## Supplementary Material

Supplementary Table 1 Clinical data in TCGA LIHC mRNA data.

Supplementary Table 2 Clinical data in TCGA LIHC miRNA data.
