# Supplementary figures and images for: Long Noncoding RNA SNHG1 Regulates LMNB2 Expression by Sponging miR-326 and Promotes Cancer Growth in Hepatocellular Carcinoma
Source: Front Oncol. 2021 Nov 30;11:784067. doi: 10.3389/fonc.2021.784067 (PMC8670182; doi:10.3389/fonc.2021.784067)

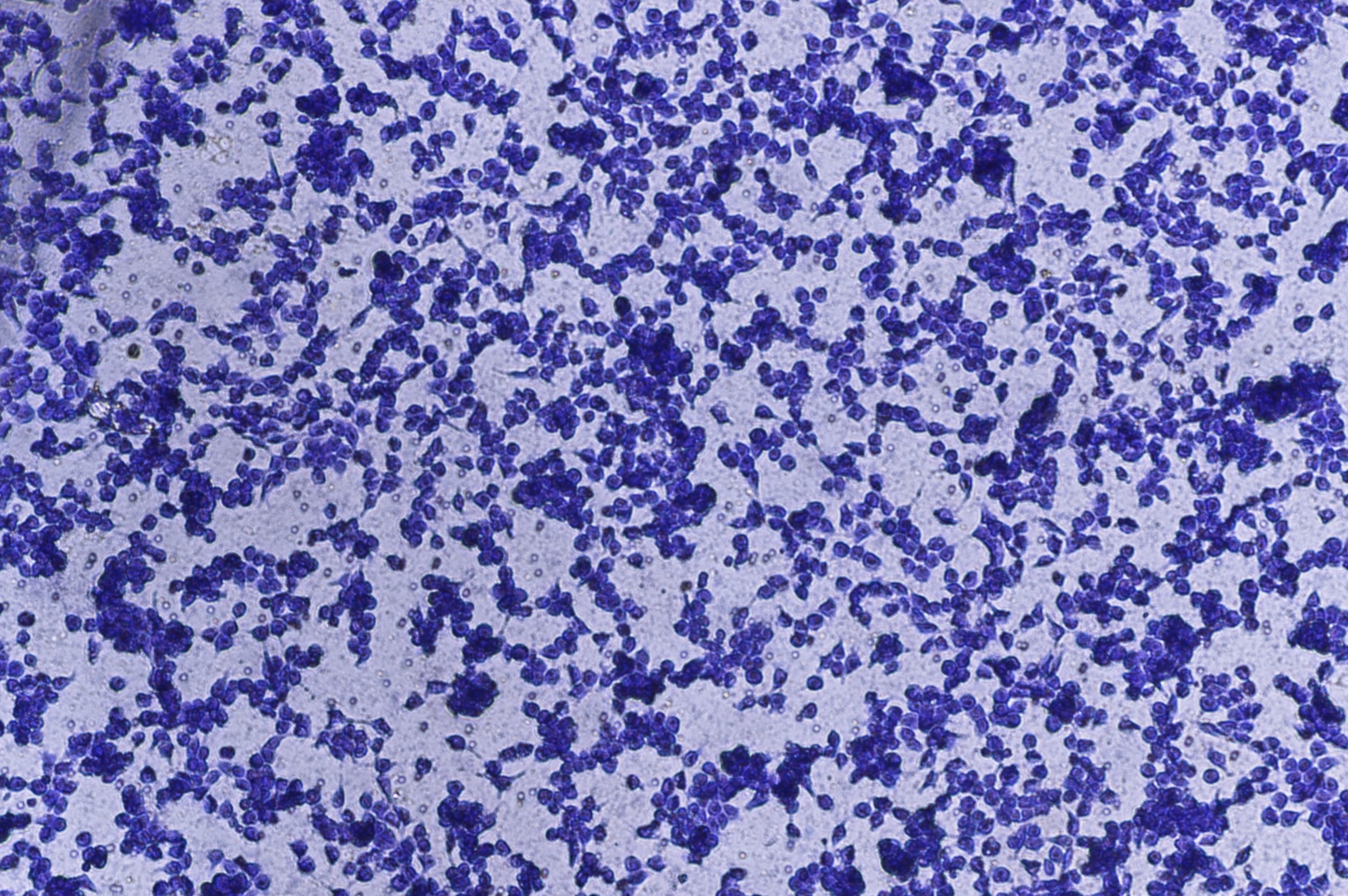

Supplement: Supplementary file 1 [file DataSheet_1.zip › source data1/1.tif]

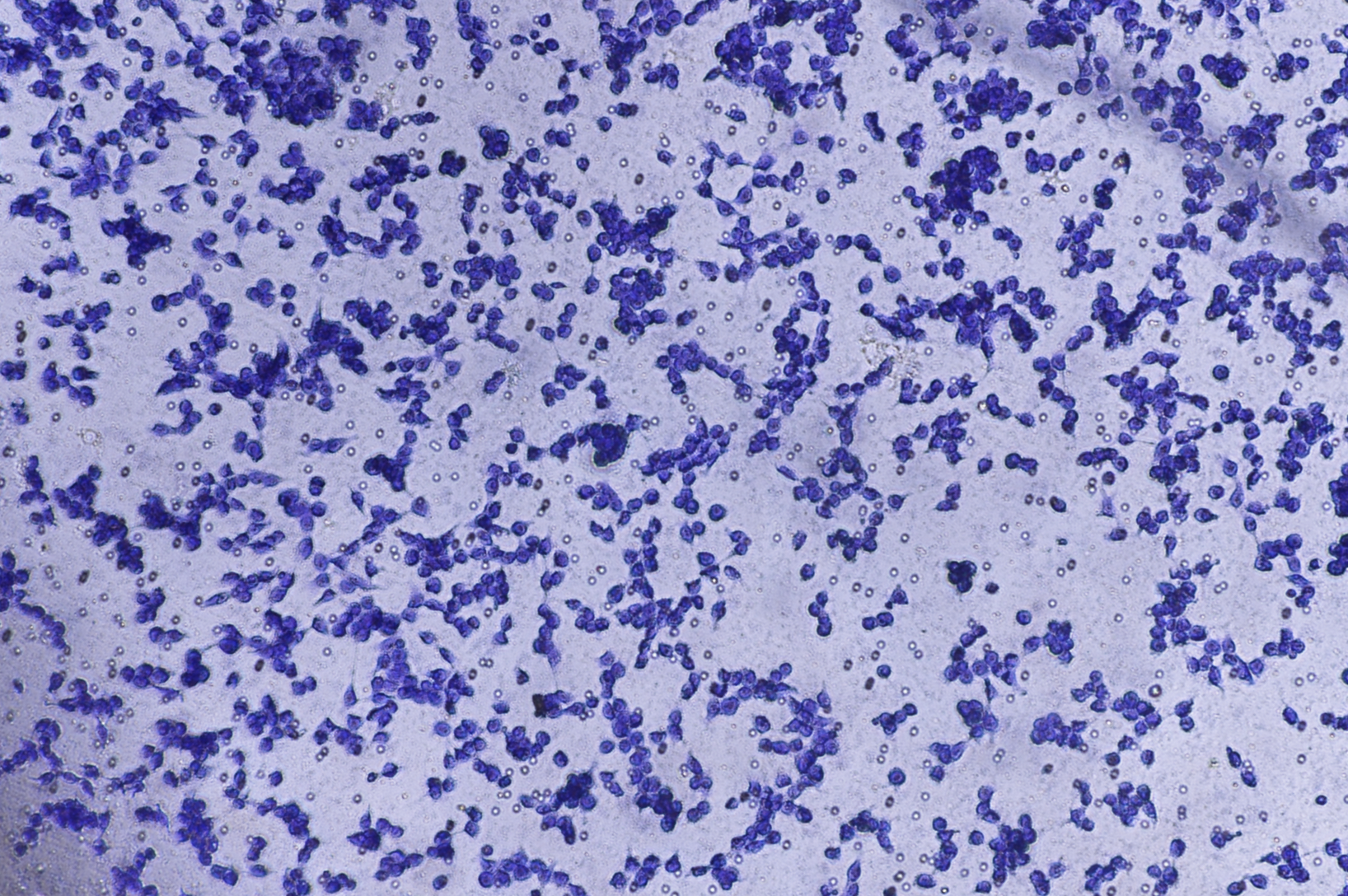

Supplement: Supplementary file 1 [file DataSheet_1.zip › source data1/2.tif]

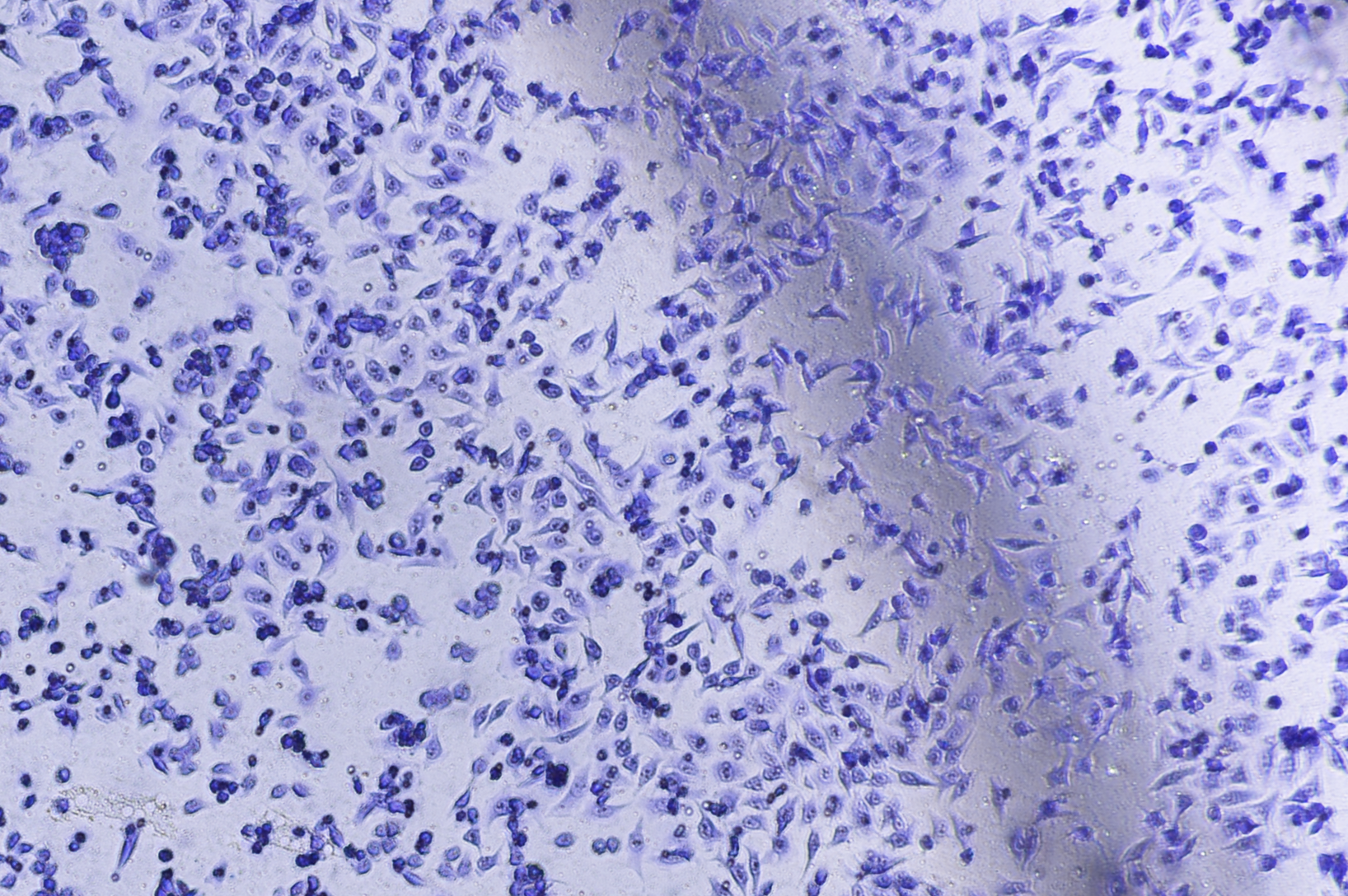

Supplement: Supplementary file 1 [file DataSheet_1.zip › source data1/3.tif]

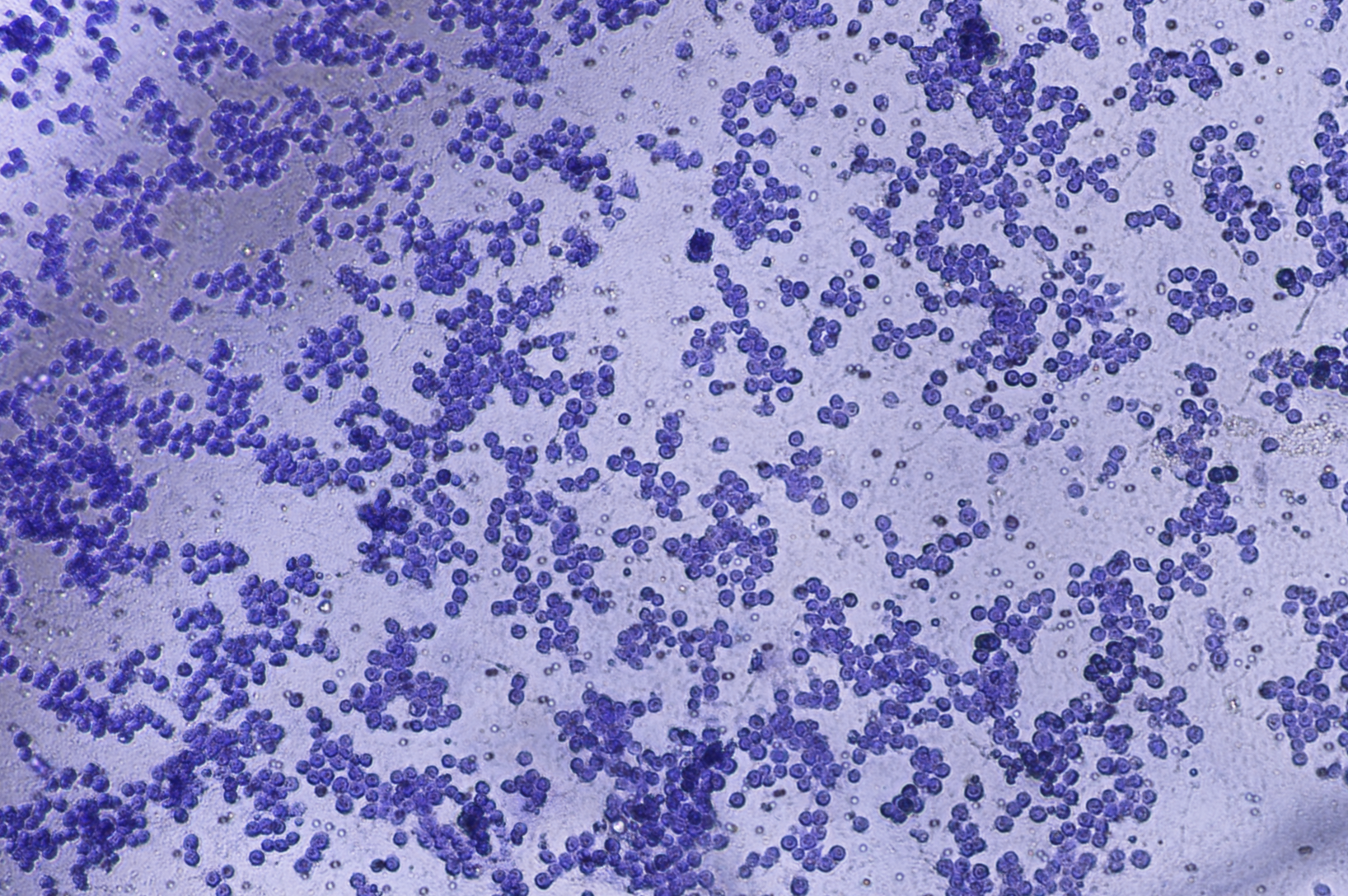

Supplement: Supplementary file 1 [file DataSheet_1.zip › source data1/4.tif]

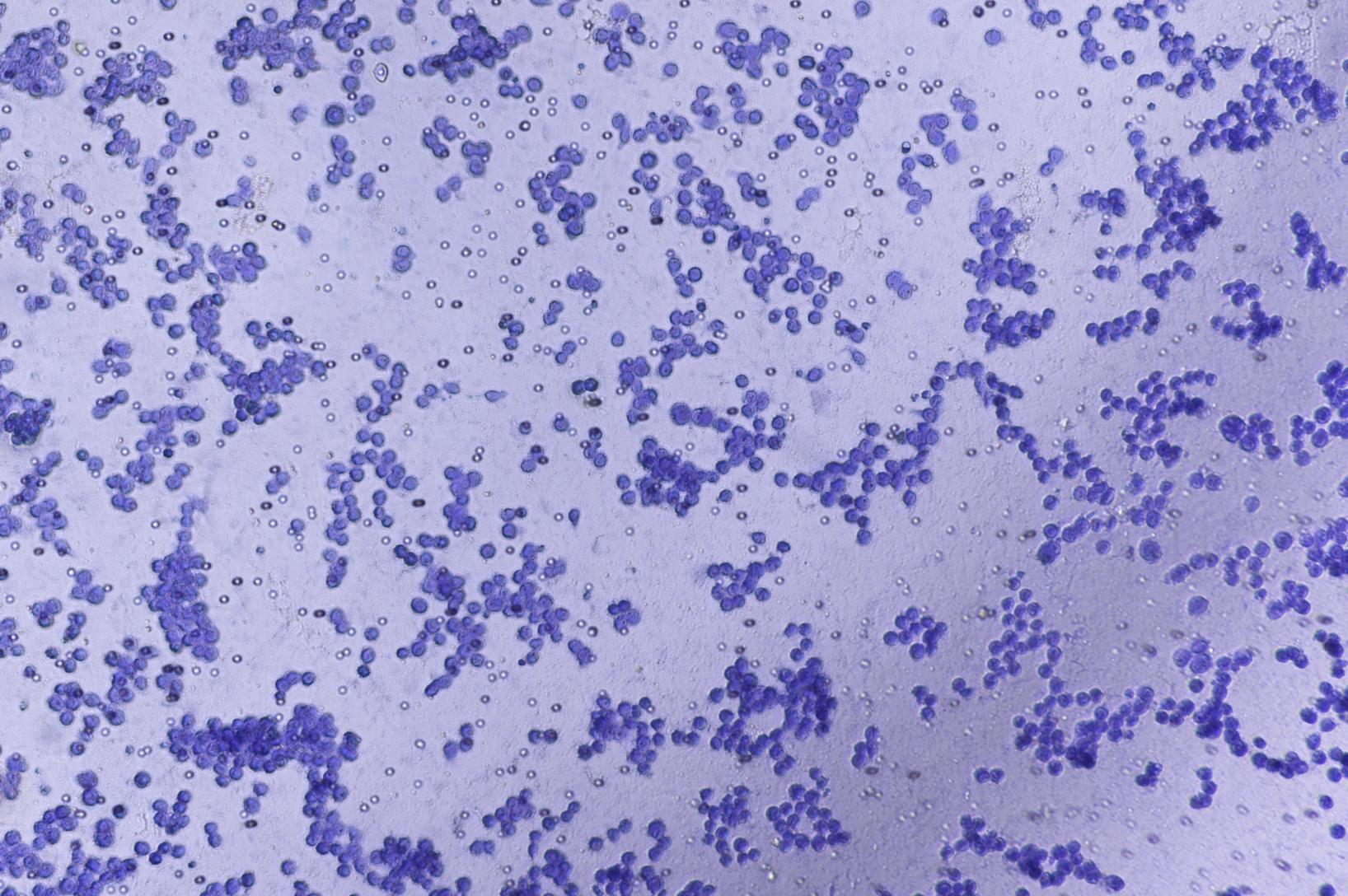

Supplement: Supplementary file 1 [file DataSheet_1.zip › source data1/5.tif]

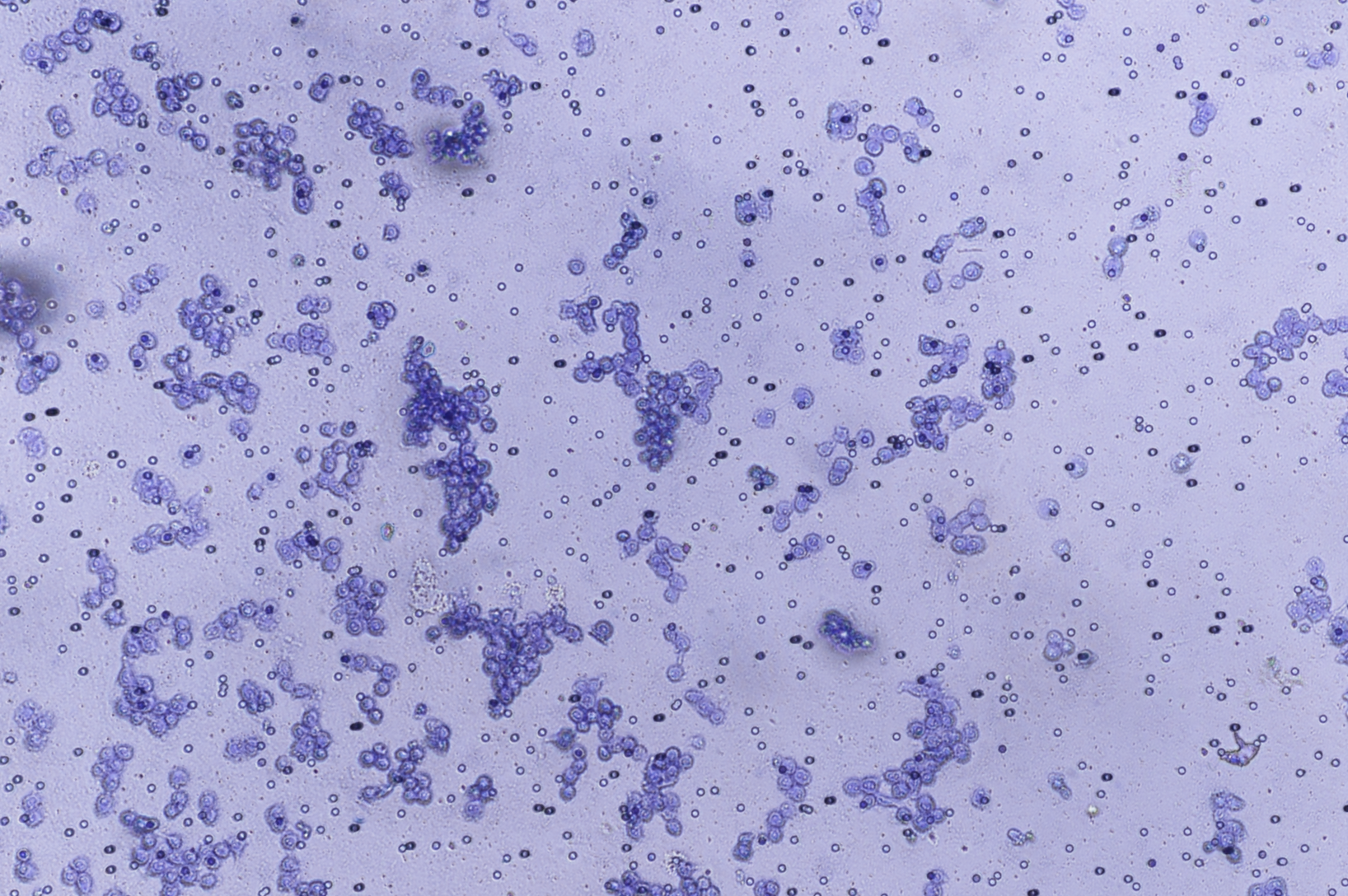

Supplement: Supplementary file 1 [file DataSheet_1.zip › source data1/6.tif]

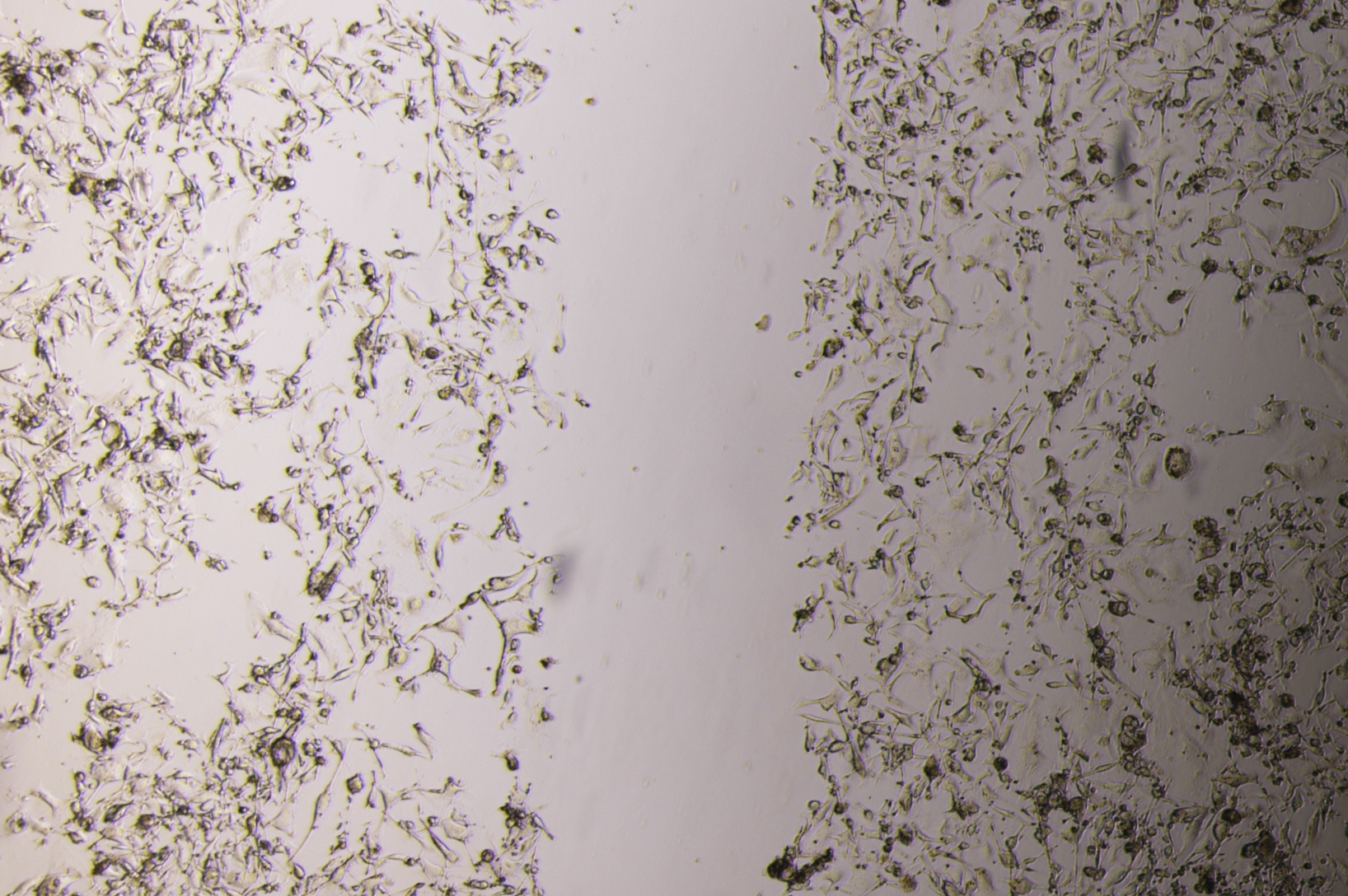

Supplement: Supplementary file 2 [file DataSheet_2.zip › source data3/31.tif]

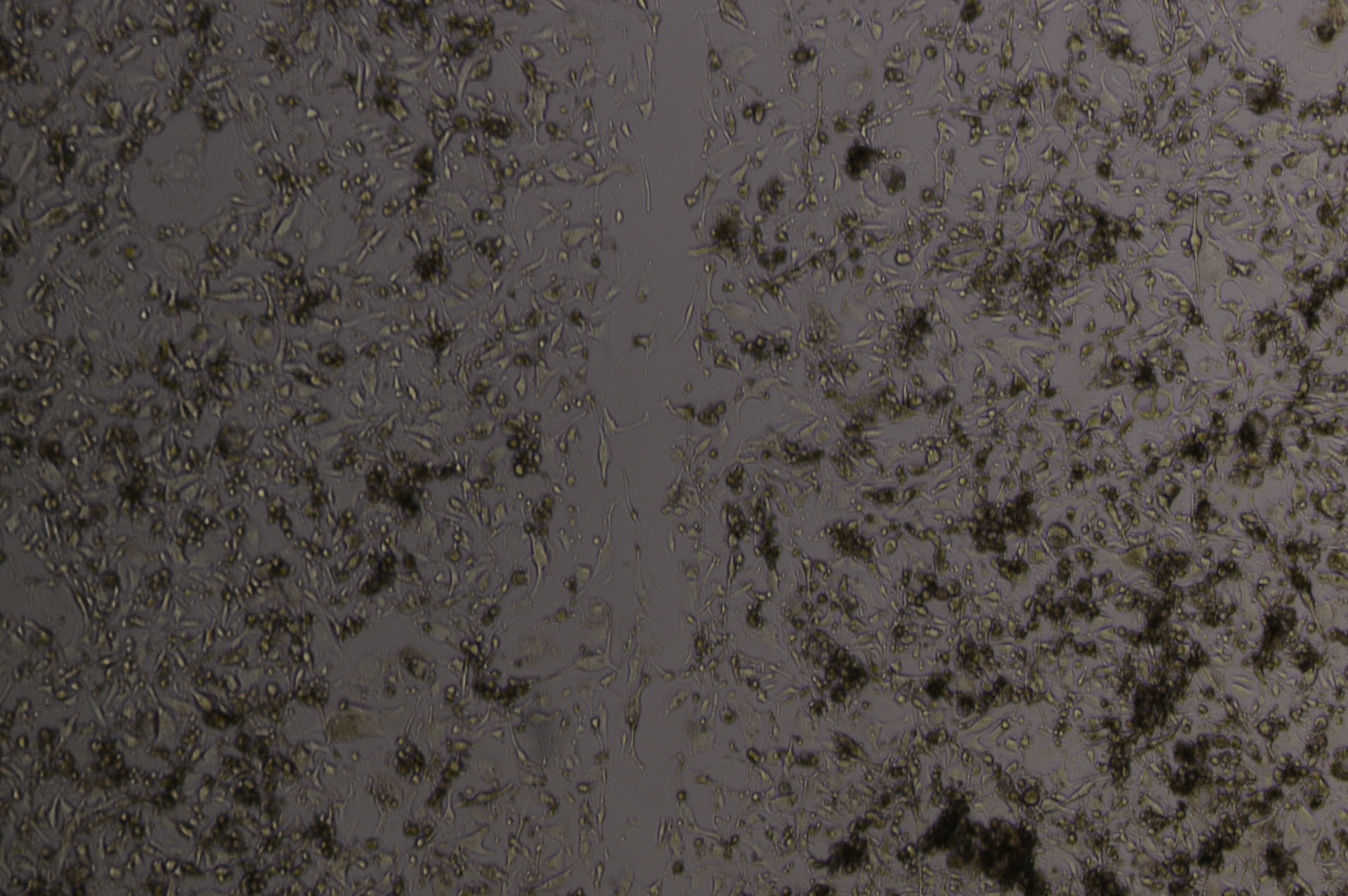

Supplement: Supplementary file 2 [file DataSheet_2.zip › source data3/32.tif]

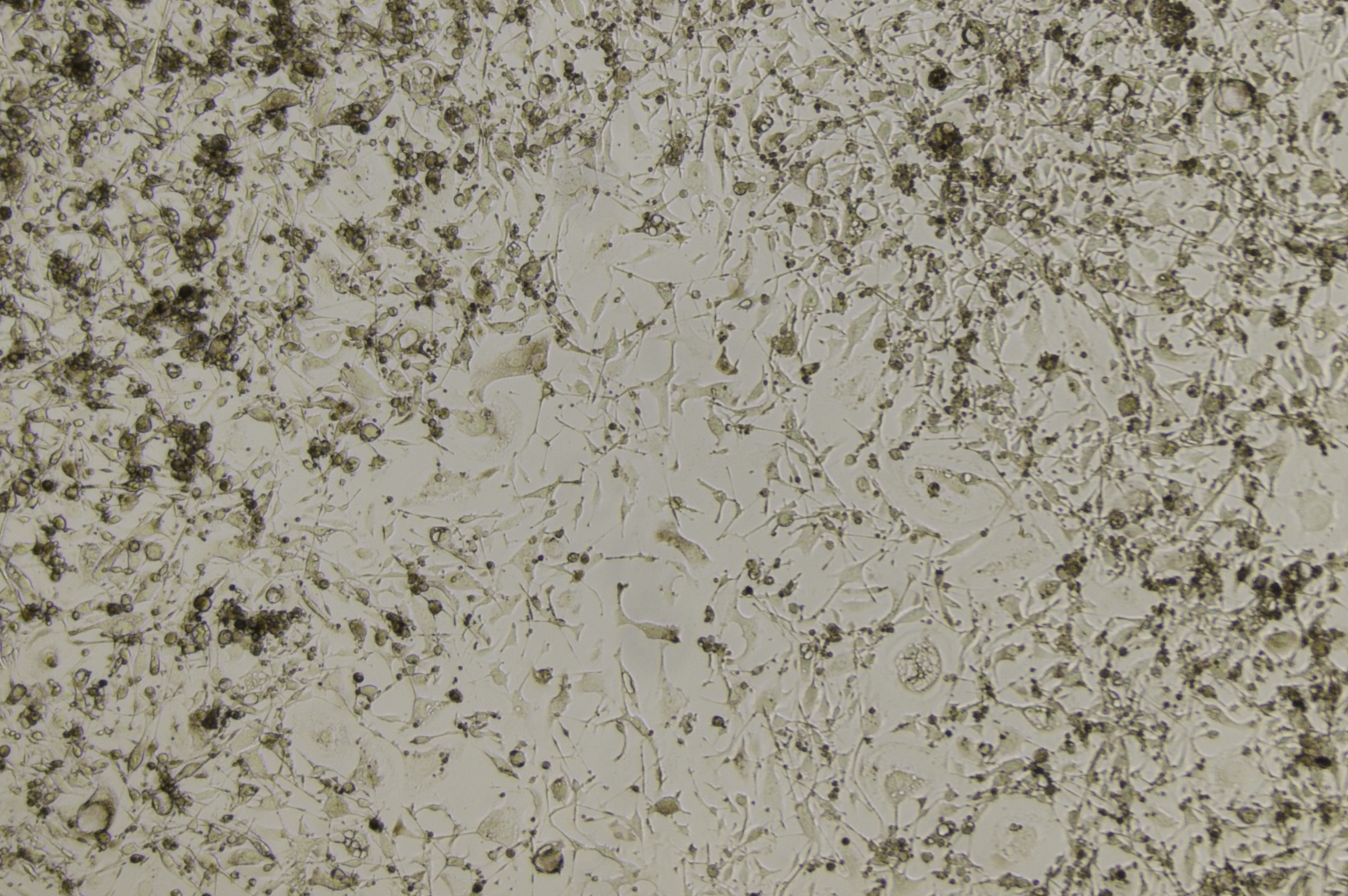

Supplement: Supplementary file 2 [file DataSheet_2.zip › source data3/33.tif]

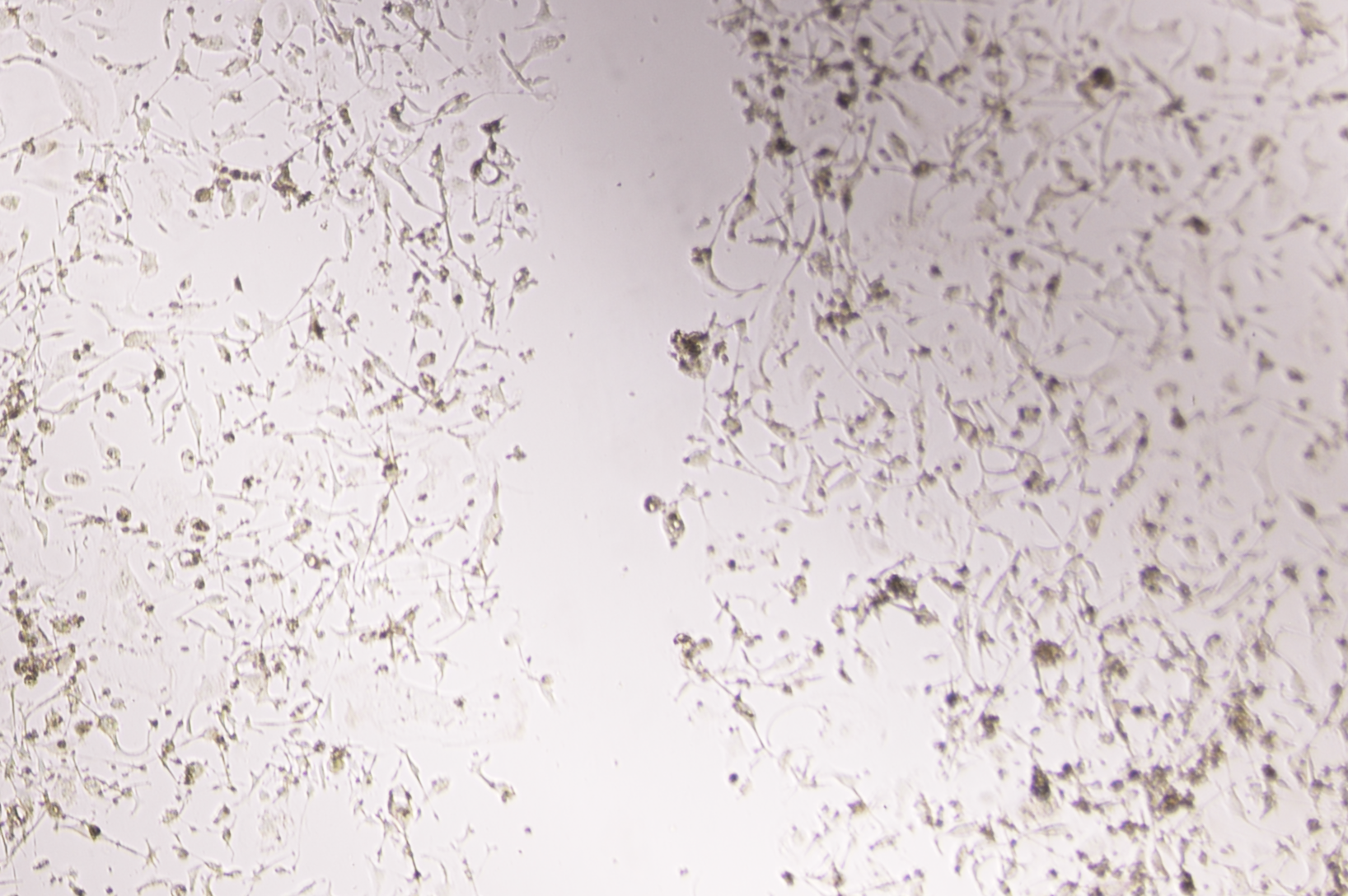

Supplement: Supplementary file 2 [file DataSheet_2.zip › source data3/34.tif]

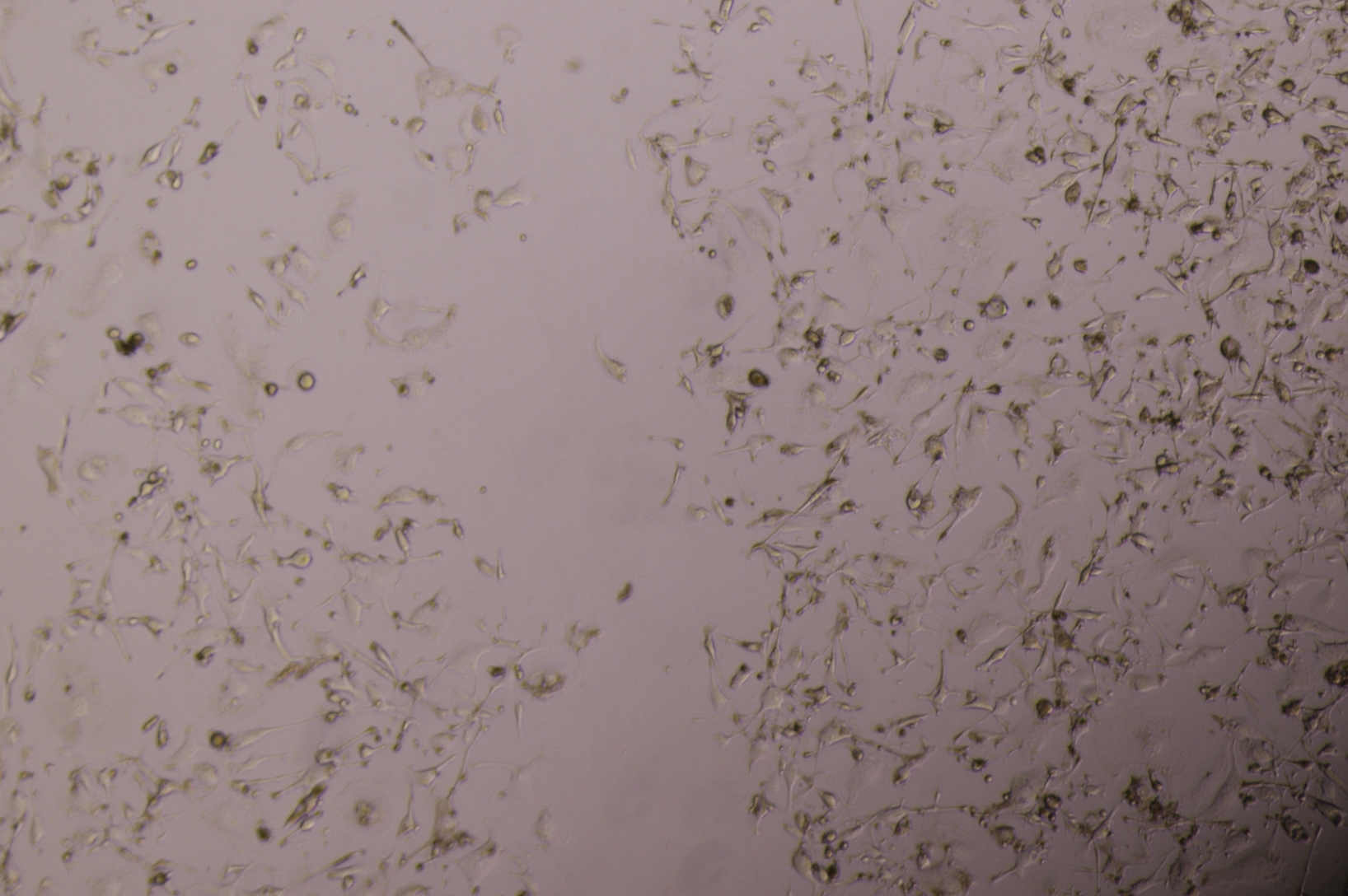

Supplement: Supplementary file 2 [file DataSheet_2.zip › source data3/35.tif]

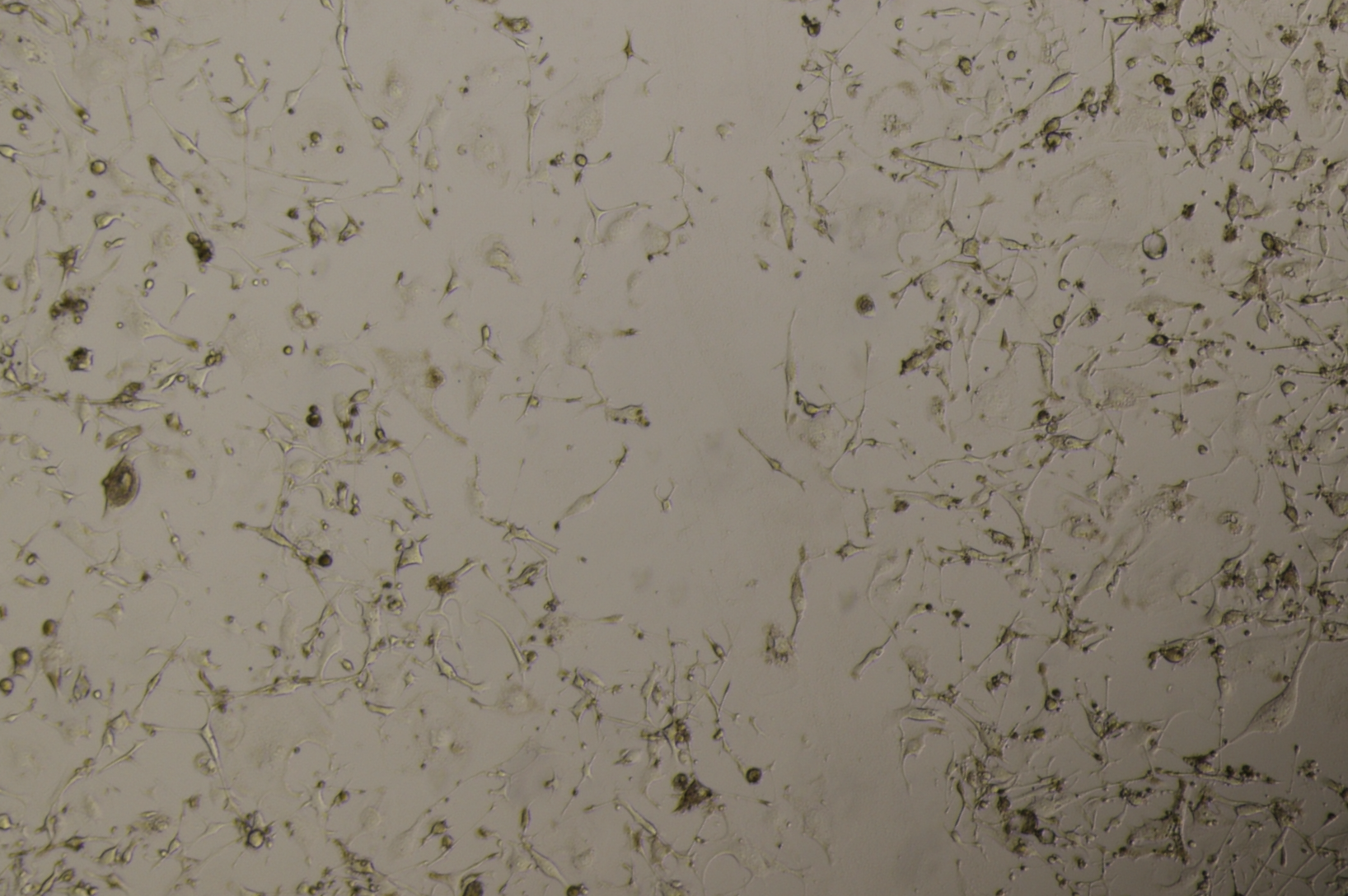

Supplement: Supplementary file 2 [file DataSheet_2.zip › source data3/36.tif]

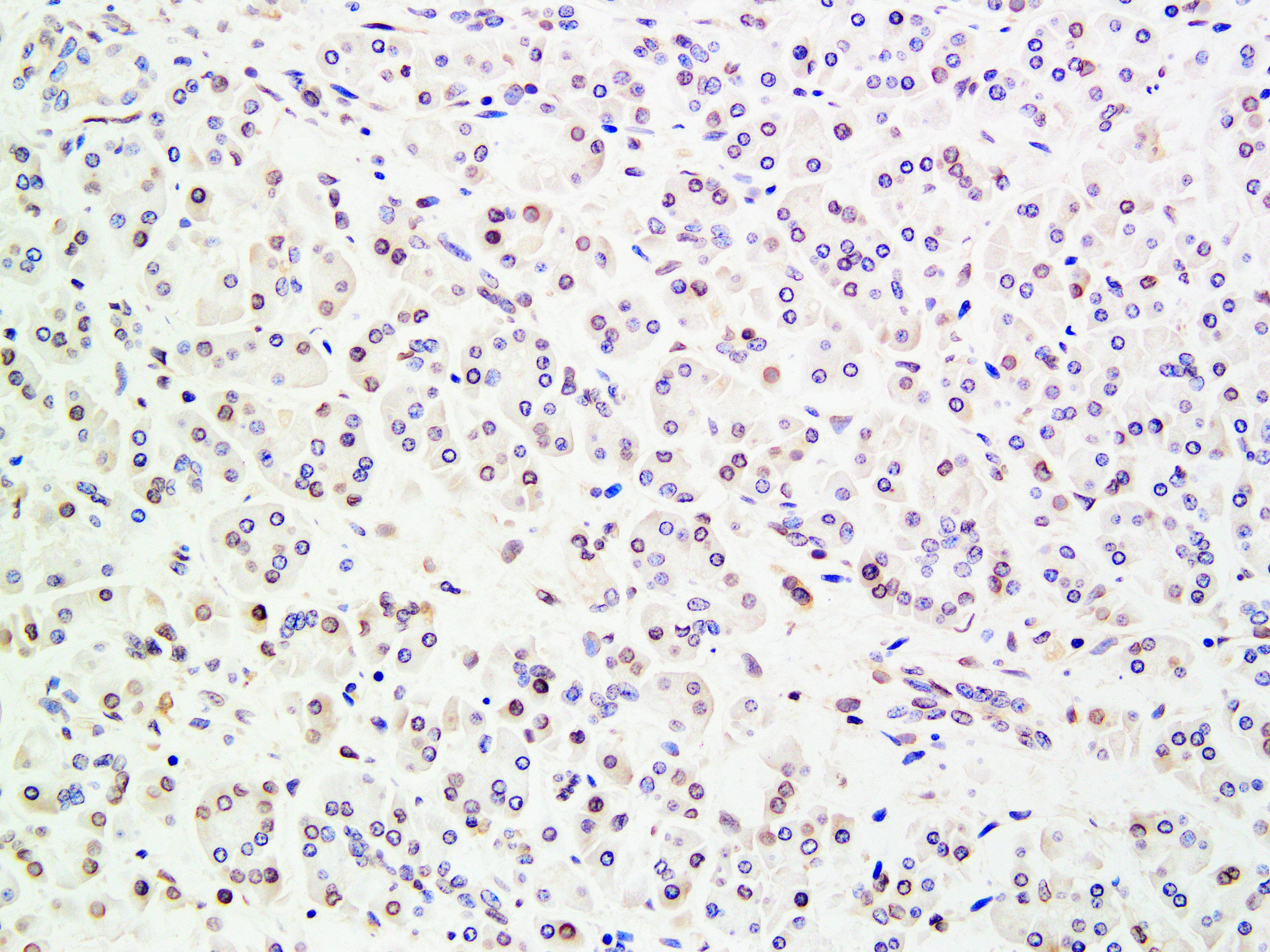

Supplement: Supplementary file 3 [file DataSheet_3.zip › source data6/high new.jpg]

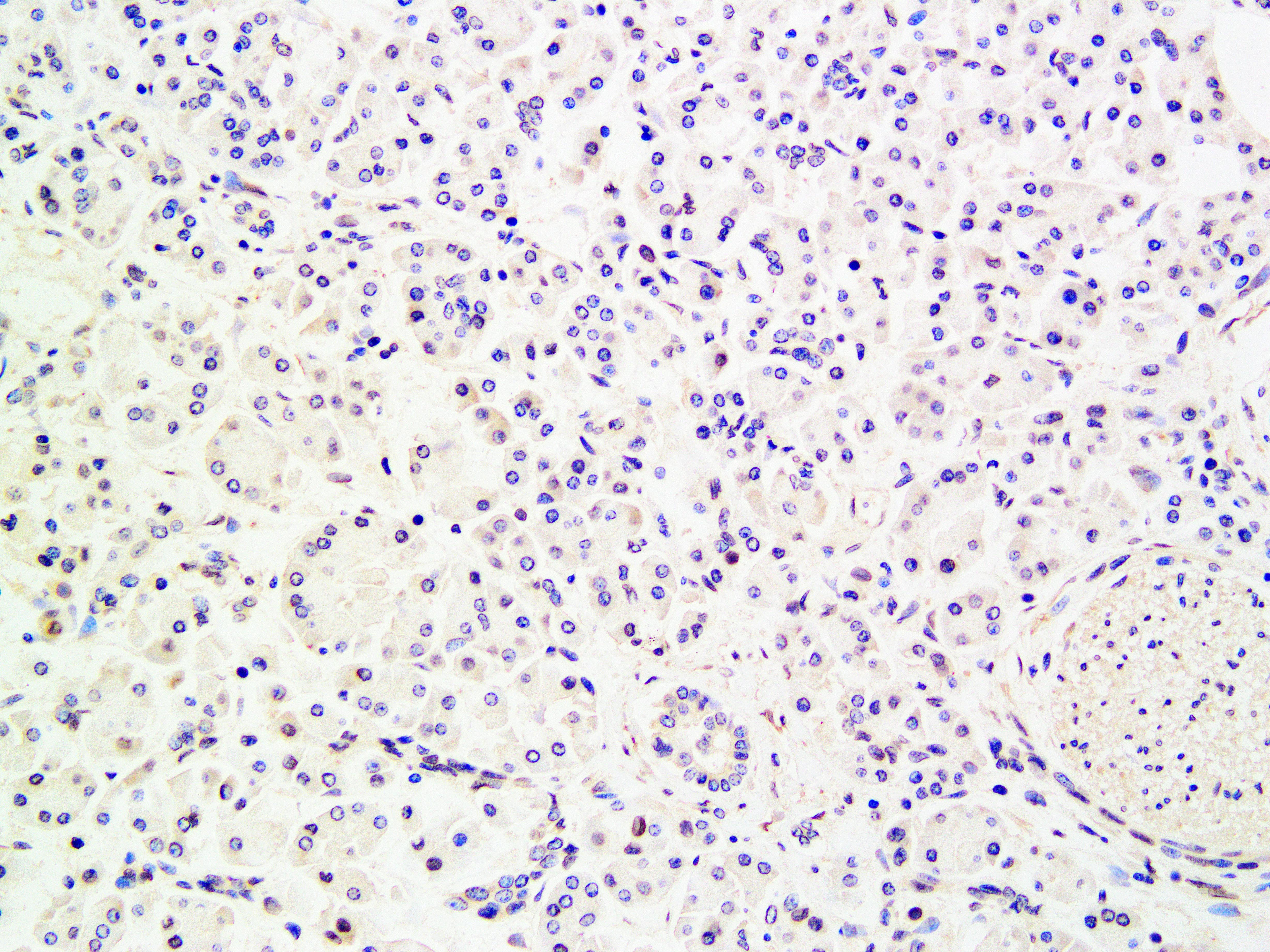

Supplement: Supplementary file 3 [file DataSheet_3.zip › source data6/low new.jpg]

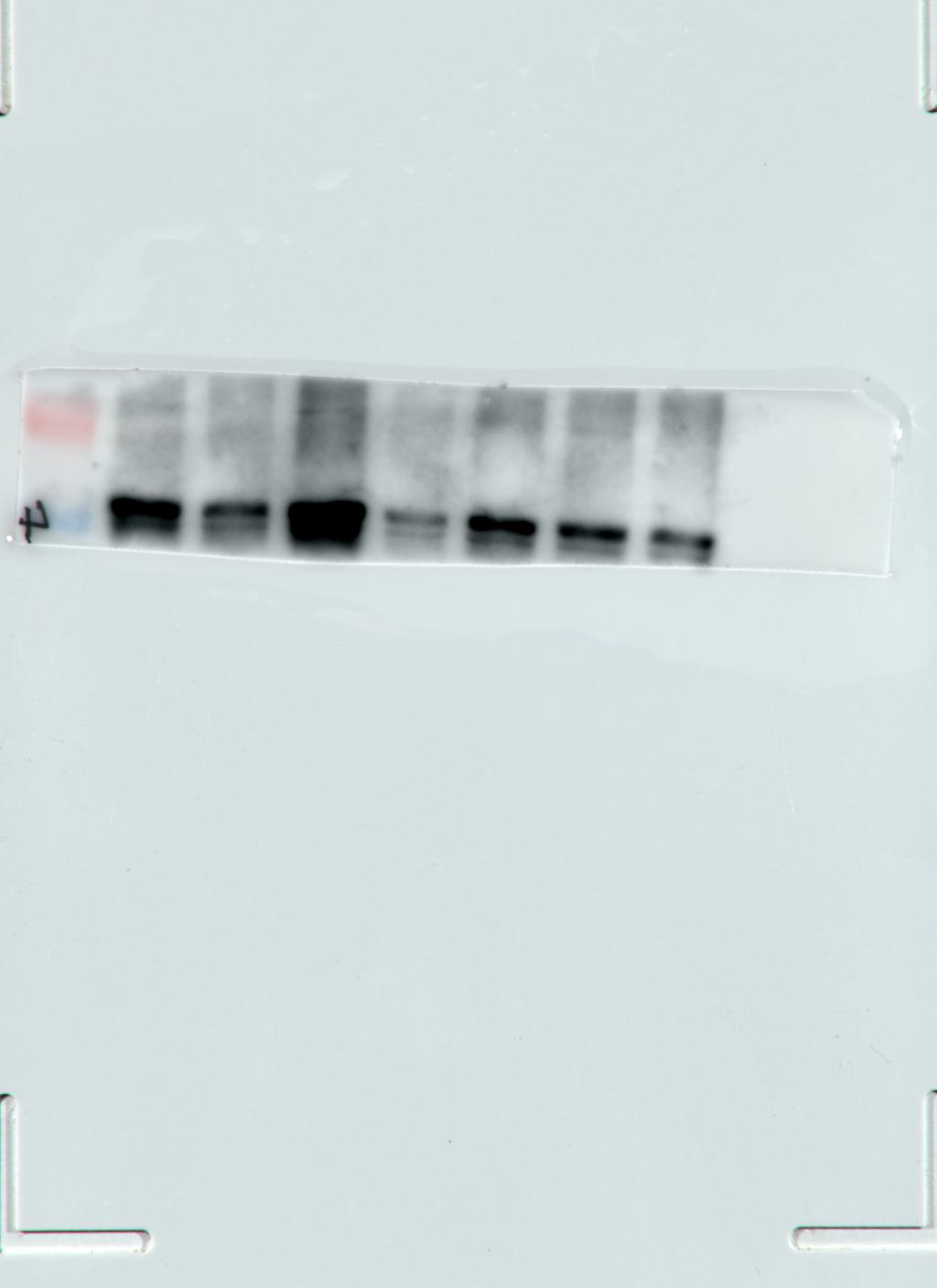

Supplement: Supplementary file 3 [file DataSheet_3.zip › source data6/mwt20200720-shmt2 22 2020.07.20_22.48.58_Ch+Marker.jpg]

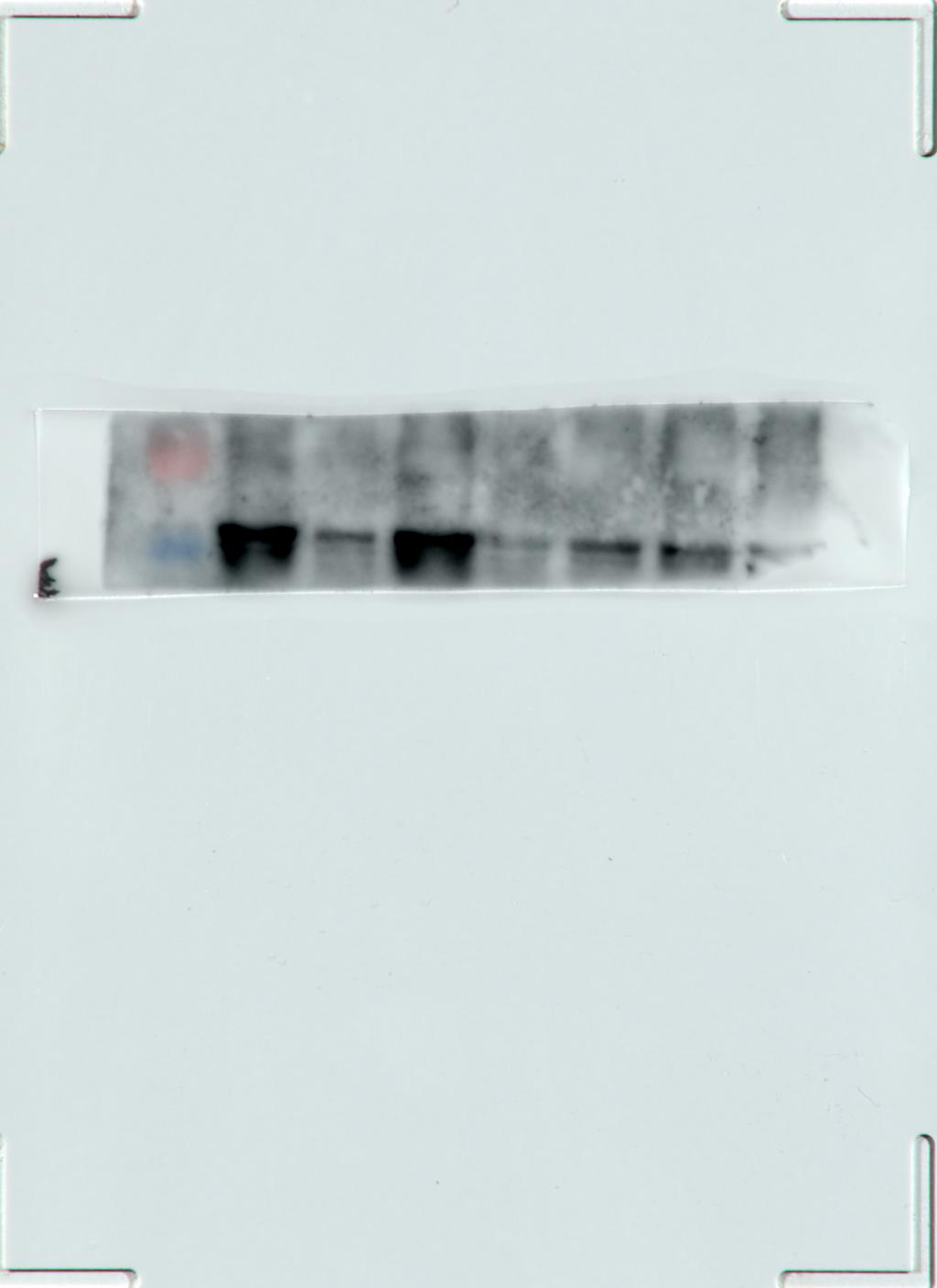

Supplement: Supplementary file 3 [file DataSheet_3.zip › source data6/mwt20200720-shmt2 2020.07.20_22.44.52_Ch+Marker.jpg]

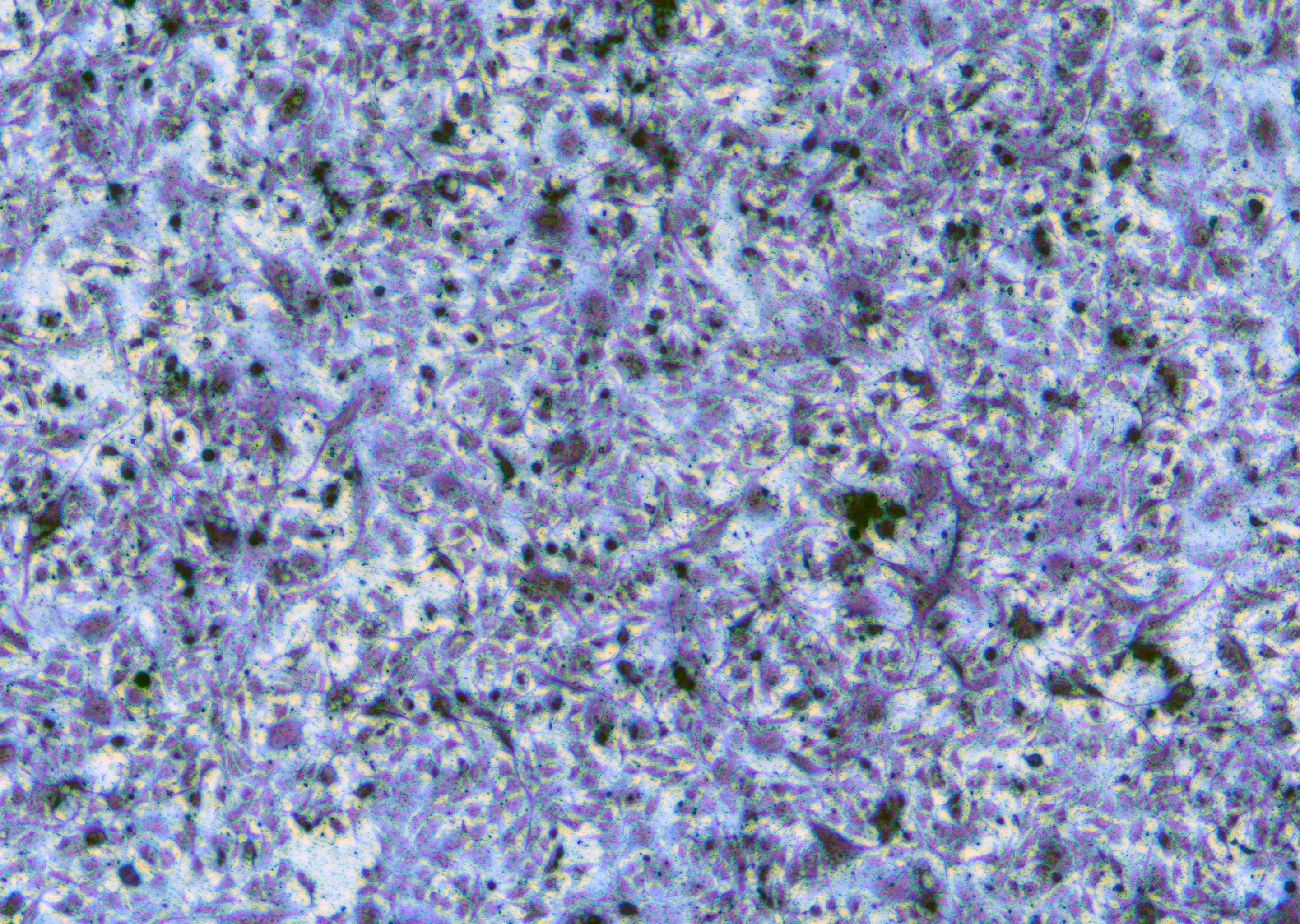

Supplement: Supplementary file 4 [file Image_1.tif]

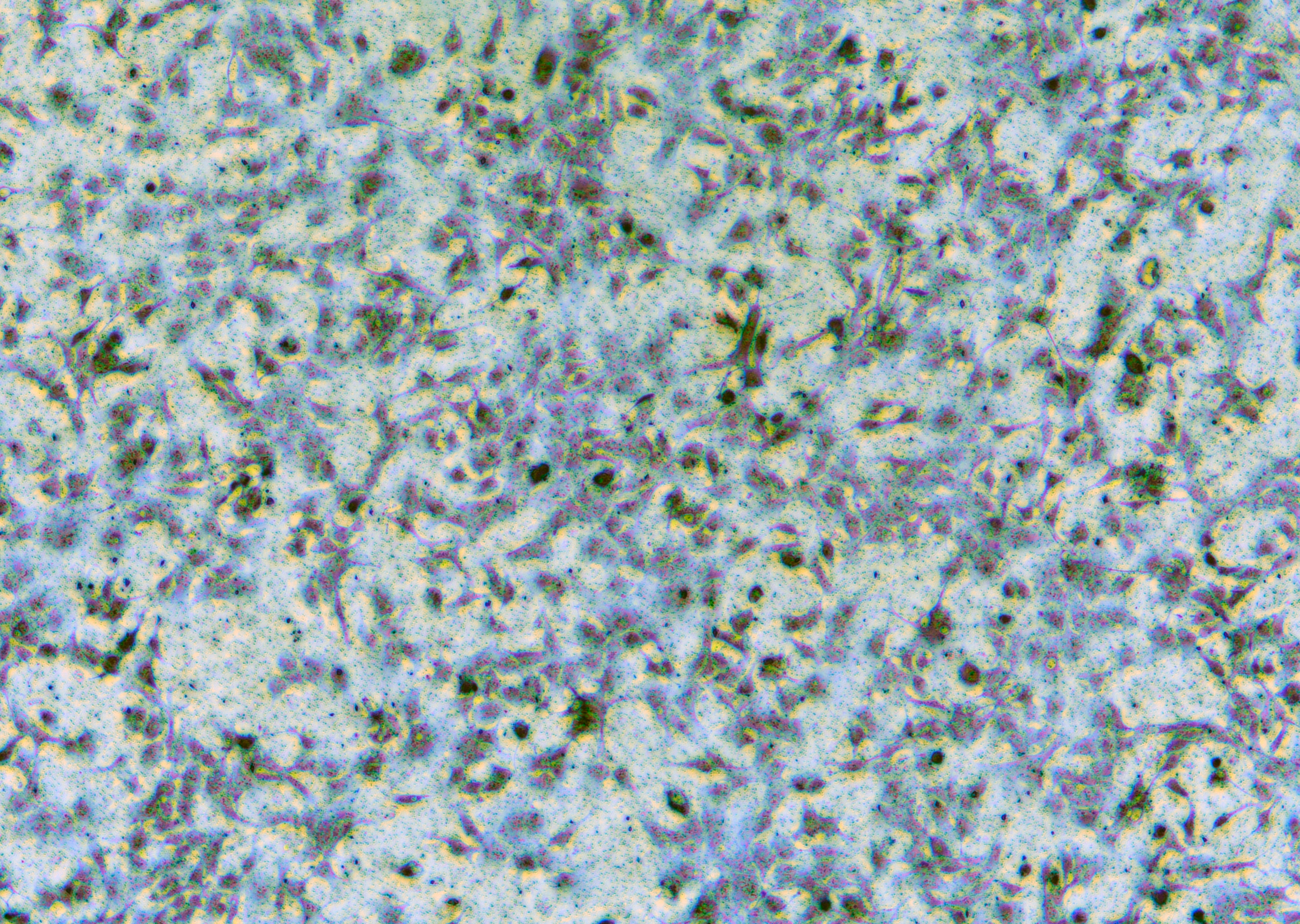

Supplement: Supplementary file 5 [file Image_2.tif]

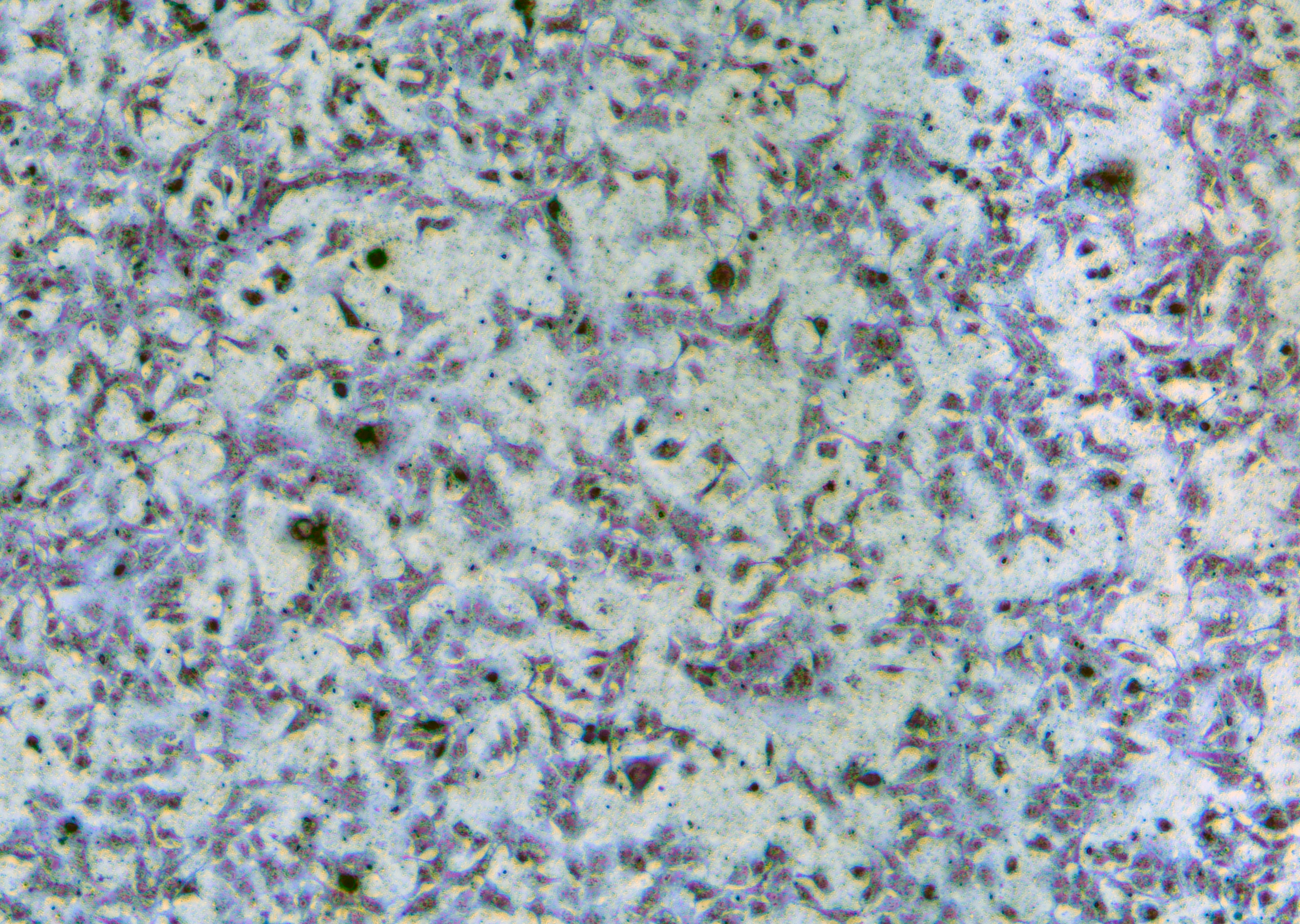

Supplement: Supplementary file 6 [file Image_3.tif]

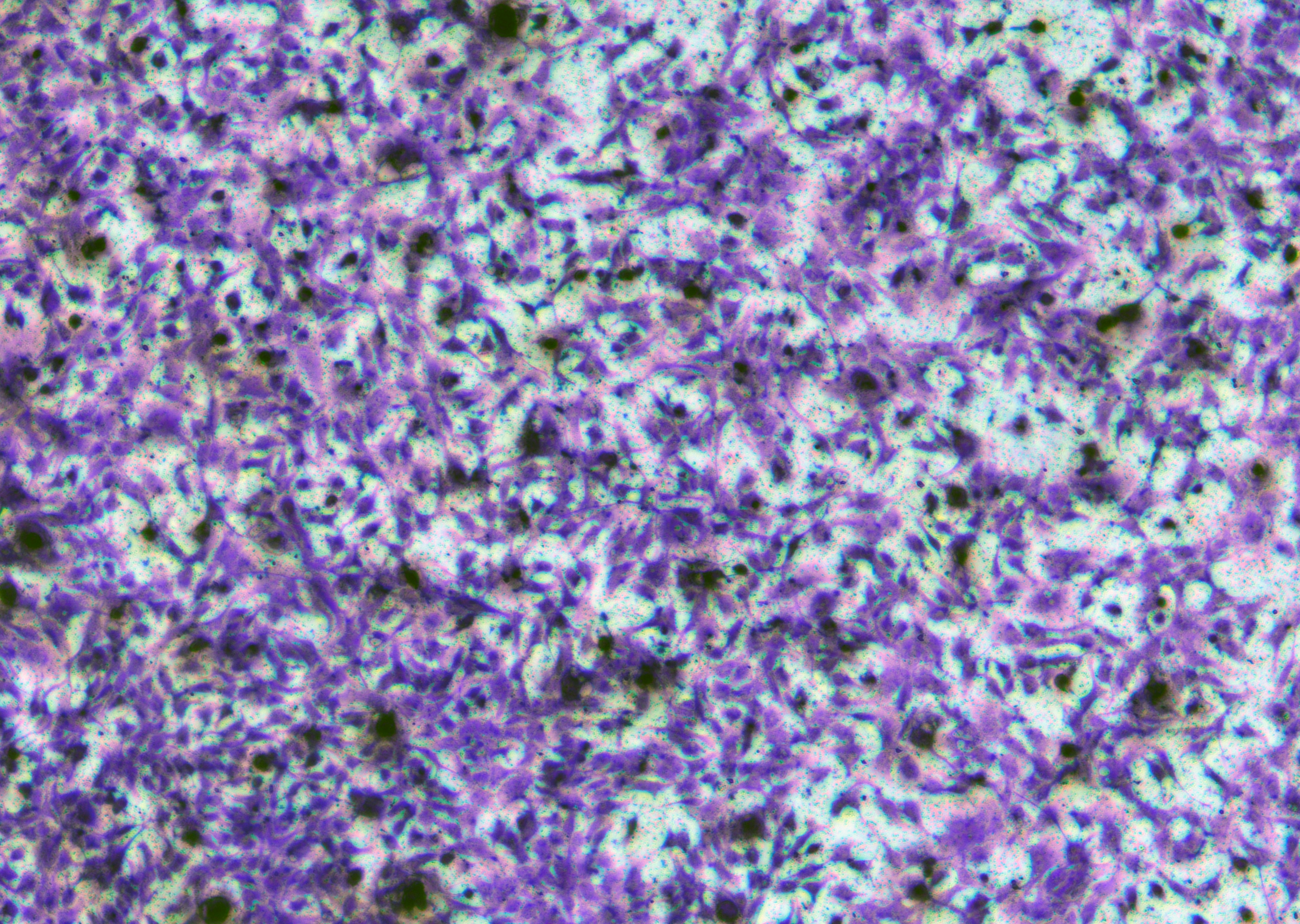

Supplement: Supplementary file 7 [file Image_4.tif]

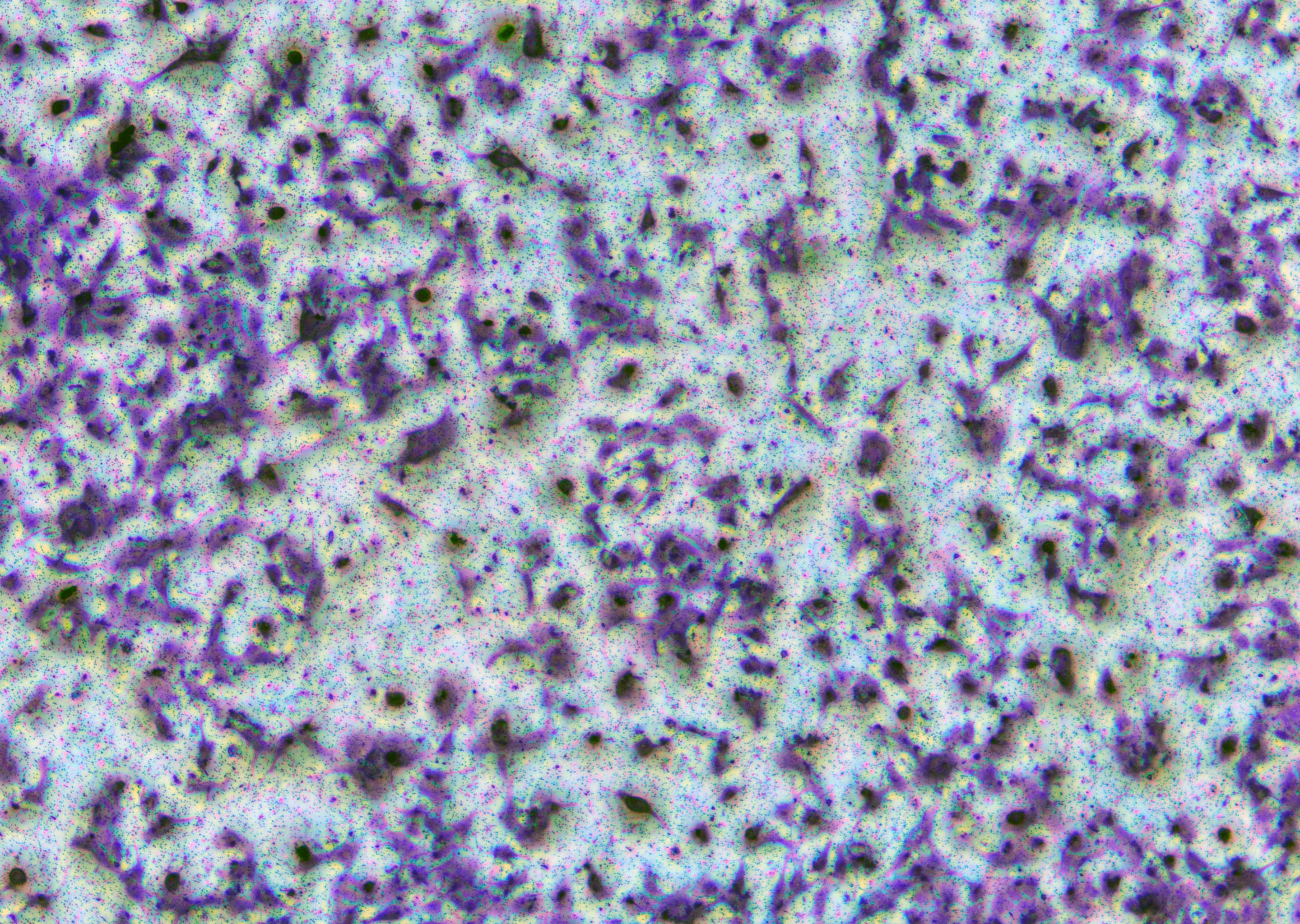

Supplement: Supplementary file 8 [file Image_5.tif]

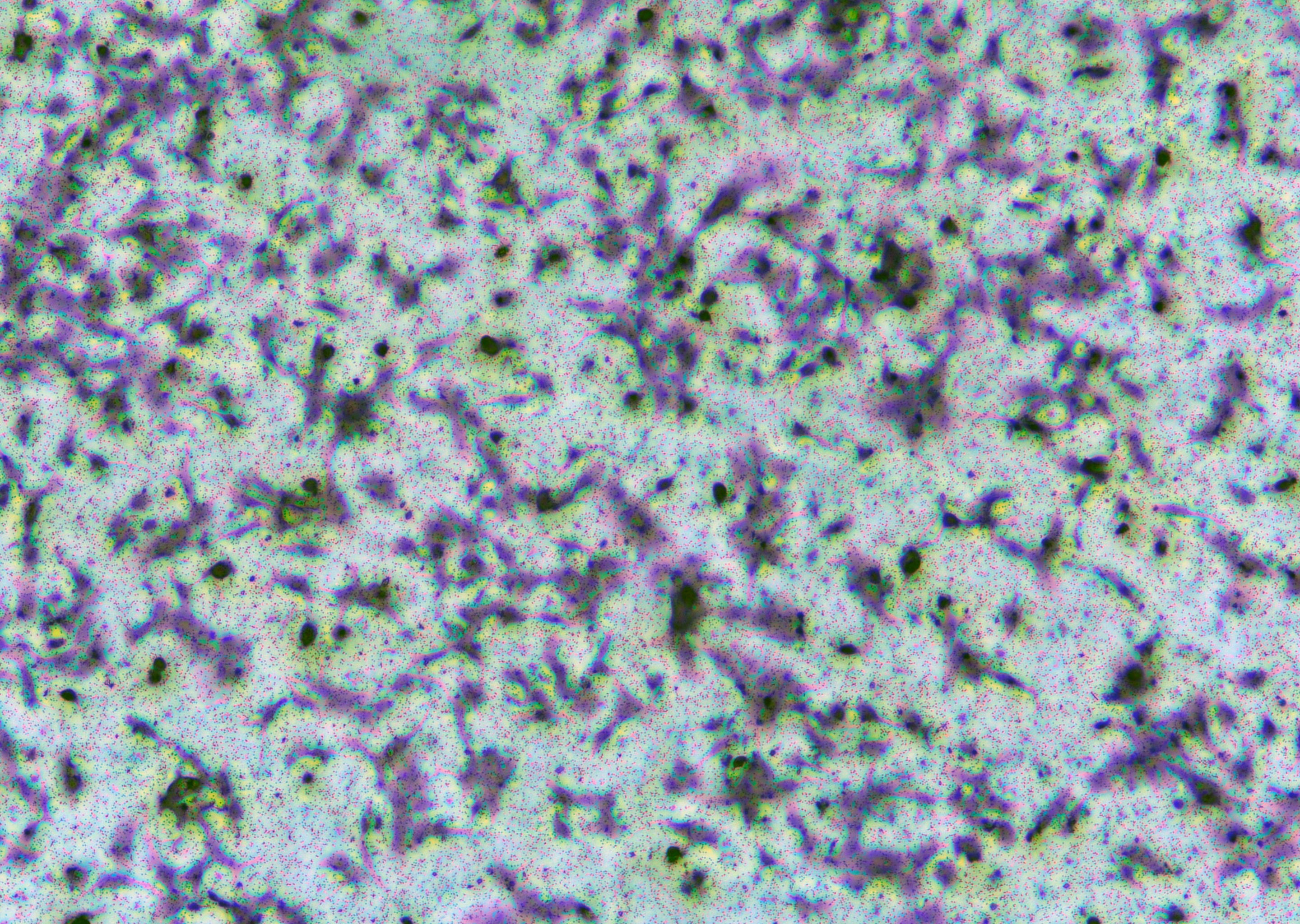

Supplement: Supplementary file 9 [file Image_6.tif]
