# Supplementary material for: Long Noncoding RNA SNHG1 Regulates LMNB2 Expression by Sponging miR-326 and Promotes Cancer Growth in Hepatocellular Carcinoma
Source: Front Oncol. 2021 Nov 30;11:784067. doi: 10.3389/fonc.2021.784067 (PMC8670182; doi:10.3389/fonc.2021.784067)
Supplement: Supplementary Table 1 — Clinical data in TCGA LIHC mRNA data. [file Table_1.docx]

|  | Project id | Case id | Sample | Sample type | Days to death | Days to last follow up |
| --- | --- | --- | --- | --- | --- | --- |
| 1 | TCGA-LIHC | TCGA-2Y-A9GX | TCGA-2Y-A9GX-01A | PrimaryTumor | NA | 2442 |
| 2 | TCGA-LIHC | TCGA-DD-A4ND | TCGA-DD-A4ND-01A | PrimaryTumor | NA | 2746 |
| 3 | TCGA-LIHC | TCGA-CC-A8HS | TCGA-CC-A8HS-01A | PrimaryTumor | 300 | NA |
| 4 | TCGA-LIHC | TCGA-CC-5259 | TCGA-CC-5259-01A | PrimaryTumor | NA | 250 |
| 5 | TCGA-LIHC | TCGA-DD-AAE4 | TCGA-DD-AAE4-01A | PrimaryTumor | NA | 608 |
| 6 | TCGA-LIHC | TCGA-ZP-A9CY | TCGA-ZP-A9CY-01A | PrimaryTumor | NA | 782 |
| 7 | TCGA-LIHC | TCGA-DD-AAW1 | TCGA-DD-AAW1-01A | PrimaryTumor | NA | 1989 |
| 8 | TCGA-LIHC | TCGA-EP-A2KC | TCGA-EP-A2KC-01A | PrimaryTumor | 19 | NA |
| 9 | TCGA-LIHC | TCGA-G3-AAV4 | TCGA-G3-AAV4-01A | PrimaryTumor | 27 | NA |
| 10 | TCGA-LIHC | TCGA-DD-A4NF | TCGA-DD-A4NF-01A | PrimaryTumor | NA | 942 |
| 11 | TCGA-LIHC | TCGA-BC-A10X | TCGA-BC-A10X-11A | SolidTissueNormal | 770 | NA |
| 12 | TCGA-LIHC | TCGA-G3-A25X | TCGA-G3-A25X-01A | PrimaryTumor | NA | 1779 |
| 13 | TCGA-LIHC | TCGA-DD-A3A1 | TCGA-DD-A3A1-11A | SolidTissueNormal | 233 | NA |
| 14 | TCGA-LIHC | TCGA-DD-A3A1 | TCGA-DD-A3A1-01A | PrimaryTumor | 233 | NA |
| 15 | TCGA-LIHC | TCGA-2Y-A9GU | TCGA-2Y-A9GU-01A | PrimaryTumor | NA | 1939 |
| 16 | TCGA-LIHC | TCGA-DD-AADL | TCGA-DD-AADL-01A | PrimaryTumor | NA | 636 |
| 17 | TCGA-LIHC | TCGA-BC-A10Q | TCGA-BC-A10Q-11A | SolidTissueNormal | 1135 | NA |
| 18 | TCGA-LIHC | TCGA-RC-A6M3 | TCGA-RC-A6M3-01A | PrimaryTumor | NA | NA |
| 19 | TCGA-LIHC | TCGA-UB-A7MD | TCGA-UB-A7MD-01A | PrimaryTumor | 52 | NA |
| 20 | TCGA-LIHC | TCGA-DD-A3A4 | TCGA-DD-A3A4-11A | SolidTissueNormal | 612 | NA |
| 21 | TCGA-LIHC | TCGA-DD-AAE8 | TCGA-DD-AAE8-01A | PrimaryTumor | NA | NA |
| 22 | TCGA-LIHC | TCGA-DD-A119 | TCGA-DD-A119-11A | SolidTissueNormal | 223 | NA |
| 23 | TCGA-LIHC | TCGA-DD-A4NE | TCGA-DD-A4NE-01A | PrimaryTumor | 660 | NA |
| 24 | TCGA-LIHC | TCGA-DD-AAE9 | TCGA-DD-AAE9-01A | PrimaryTumor | NA | 722 |
| 25 | TCGA-LIHC | TCGA-EP-A12J | TCGA-EP-A12J-11A | SolidTissueNormal | NA | 570 |
| 26 | TCGA-LIHC | TCGA-BC-A10Y | TCGA-BC-A10Y-01A | PrimaryTumor | 711 | NA |
| 27 | TCGA-LIHC | TCGA-DD-A1EB | TCGA-DD-A1EB-01A | PrimaryTumor | NA | 2017 |
| 28 | TCGA-LIHC | TCGA-ED-A4XI | TCGA-ED-A4XI-01A | PrimaryTumor | NA | 819 |
| 29 | TCGA-LIHC | TCGA-DD-A39X | TCGA-DD-A39X-11A | SolidTissueNormal | 1694 | NA |
| 30 | TCGA-LIHC | TCGA-G3-AAV5 | TCGA-G3-AAV5-01A | PrimaryTumor | NA | 354 |
| 31 | TCGA-LIHC | TCGA-DD-A11C | TCGA-DD-A11C-11A | SolidTissueNormal | NA | 662 |
| 32 | TCGA-LIHC | TCGA-DD-A73D | TCGA-DD-A73D-01A | PrimaryTumor | NA | 693 |
| 33 | TCGA-LIHC | TCGA-RC-A7S9 | TCGA-RC-A7S9-01A | PrimaryTumor | NA | 640 |
| 34 | TCGA-LIHC | TCGA-DD-AAE7 | TCGA-DD-AAE7-01A | PrimaryTumor | NA | 644 |
| 35 | TCGA-LIHC | TCGA-LG-A9QC | TCGA-LG-A9QC-01A | PrimaryTumor | NA | 425 |
| 36 | TCGA-LIHC | TCGA-ZS-A9CE | TCGA-ZS-A9CE-01A | PrimaryTumor | NA | 1241 |
| 37 | TCGA-LIHC | TCGA-ED-A7PZ | TCGA-ED-A7PZ-01A | PrimaryTumor | NA | 6 |
| 38 | TCGA-LIHC | TCGA-DD-A1EE | TCGA-DD-A1EE-01A | PrimaryTumor | 349 | 204 |
| 39 | TCGA-LIHC | TCGA-G3-A5SM | TCGA-G3-A5SM-01A | PrimaryTumor | NA | 520 |
| 40 | TCGA-LIHC | TCGA-DD-A3A4 | TCGA-DD-A3A4-01A | PrimaryTumor | 612 | NA |
| 41 | TCGA-LIHC | TCGA-ED-A7PX | TCGA-ED-A7PX-01A | PrimaryTumor | NA | 6 |
| 42 | TCGA-LIHC | TCGA-DD-A1EH | TCGA-DD-A1EH-01A | PrimaryTumor | NA | 1495 |
| 43 | TCGA-LIHC | TCGA-DD-A73E | TCGA-DD-A73E-01A | PrimaryTumor | NA | 44 |
| 44 | TCGA-LIHC | TCGA-DD-A3A7 | TCGA-DD-A3A7-01A | PrimaryTumor | 419 | NA |
| 45 | TCGA-LIHC | TCGA-BC-A10W | TCGA-BC-A10W-01A | PrimaryTumor | 91 | NA |
| 46 | TCGA-LIHC | TCGA-CC-A5UD | TCGA-CC-A5UD-01A | PrimaryTumor | 304 | NA |
| 47 | TCGA-LIHC | TCGA-BC-A110 | TCGA-BC-A110-11A | SolidTissueNormal | 2116 | NA |
| 48 | TCGA-LIHC | TCGA-PD-A5DF | TCGA-PD-A5DF-01A | PrimaryTumor | 639 | NA |
| 49 | TCGA-LIHC | TCGA-EP-A3JL | TCGA-EP-A3JL-01A | PrimaryTumor | NA | 303 |
| 50 | TCGA-LIHC | TCGA-5C-A9VG | TCGA-5C-A9VG-01A | PrimaryTumor | NA | 328 |
| 51 | TCGA-LIHC | TCGA-DD-AAW0 | TCGA-DD-AAW0-01A | PrimaryTumor | NA | 2015 |
| 52 | TCGA-LIHC | TCGA-ZP-A9D2 | TCGA-ZP-A9D2-01A | PrimaryTumor | 765 | 743 |
| 53 | TCGA-LIHC | TCGA-CC-A3MA | TCGA-CC-A3MA-01A | PrimaryTumor | 303 | 2 |
| 54 | TCGA-LIHC | TCGA-BC-A110 | TCGA-BC-A110-01A | PrimaryTumor | 2116 | NA |
| 55 | TCGA-LIHC | TCGA-DD-A73C | TCGA-DD-A73C-01A | PrimaryTumor | NA | 701 |
| 56 | TCGA-LIHC | TCGA-CC-5258 | TCGA-CC-5258-01A | PrimaryTumor | 129 | NA |
| 57 | TCGA-LIHC | TCGA-RG-A7D4 | TCGA-RG-A7D4-01A | PrimaryTumor | NA | 1098 |
| 58 | TCGA-LIHC | TCGA-MI-A75C | TCGA-MI-A75C-01A | PrimaryTumor | NA | 291 |
| 59 | TCGA-LIHC | TCGA-DD-A114 | TCGA-DD-A114-01A | PrimaryTumor | 1149 | NA |
| 60 | TCGA-LIHC | TCGA-DD-AACO | TCGA-DD-AACO-01A | PrimaryTumor | NA | 1876 |
| 61 | TCGA-LIHC | TCGA-WX-AA47 | TCGA-WX-AA47-01A | PrimaryTumor | 556 | NA |
| 62 | TCGA-LIHC | TCGA-DD-A4NG | TCGA-DD-A4NG-01A | PrimaryTumor | 802 | 445 |
| 63 | TCGA-LIHC | TCGA-ZS-A9CD | TCGA-ZS-A9CD-01A | PrimaryTumor | 1386 | NA |
| 64 | TCGA-LIHC | TCGA-DD-AAD3 | TCGA-DD-AAD3-01A | PrimaryTumor | NA | 1295 |
| 65 | TCGA-LIHC | TCGA-G3-A3CJ | TCGA-G3-A3CJ-01A | PrimaryTumor | NA | 594 |
| 66 | TCGA-LIHC | TCGA-GJ-A3OU | TCGA-GJ-A3OU-01A | PrimaryTumor | NA | NA |
| 67 | TCGA-LIHC | TCGA-DD-AADJ | TCGA-DD-AADJ-01A | PrimaryTumor | NA | 1066 |
| 68 | TCGA-LIHC | TCGA-XR-A8TC | TCGA-XR-A8TC-01A | PrimaryTumor | NA | 1339 |
| 69 | TCGA-LIHC | TCGA-DD-A4NN | TCGA-DD-A4NN-01A | PrimaryTumor | 899 | NA |
| 70 | TCGA-LIHC | TCGA-BC-A3KF | TCGA-BC-A3KF-01A | PrimaryTumor | NA | 8 |
| 71 | TCGA-LIHC | TCGA-BC-A69I | TCGA-BC-A69I-01A | PrimaryTumor | NA | 387 |
| 72 | TCGA-LIHC | TCGA-2Y-A9GZ | TCGA-2Y-A9GZ-01A | PrimaryTumor | 848 | NA |
| 73 | TCGA-LIHC | TCGA-DD-AAEG | TCGA-DD-AAEG-01A | PrimaryTumor | NA | 719 |
| 74 | TCGA-LIHC | TCGA-DD-AACU | TCGA-DD-AACU-01A | PrimaryTumor | NA | 1567 |
| 75 | TCGA-LIHC | TCGA-FV-A23B | TCGA-FV-A23B-01A | PrimaryTumor | 1852 | 987 |
| 76 | TCGA-LIHC | TCGA-BC-4073 | TCGA-BC-4073-01B | PrimaryTumor | NA | 849 |
| 77 | TCGA-LIHC | TCGA-2Y-A9H5 | TCGA-2Y-A9H5-01A | PrimaryTumor | 555 | NA |
| 78 | TCGA-LIHC | TCGA-UB-AA0U | TCGA-UB-AA0U-01A | PrimaryTumor | NA | 327 |
| 79 | TCGA-LIHC | TCGA-T1-A6J8 | TCGA-T1-A6J8-01A | PrimaryTumor | NA | 23 |
| 80 | TCGA-LIHC | TCGA-FV-A2QQ | TCGA-FV-A2QQ-01A | PrimaryTumor | NA | 729 |
| 81 | TCGA-LIHC | TCGA-YA-A8S7 | TCGA-YA-A8S7-01A | PrimaryTumor | 412 | NA |
| 82 | TCGA-LIHC | TCGA-DD-AADY | TCGA-DD-AADY-01A | PrimaryTumor | NA | 555 |
| 83 | TCGA-LIHC | TCGA-DD-A118 | TCGA-DD-A118-01A | PrimaryTumor | NA | 3437 |
| 84 | TCGA-LIHC | TCGA-FV-A2QR | TCGA-FV-A2QR-01A | PrimaryTumor | 581 | NA |
| 85 | TCGA-LIHC | TCGA-DD-AADM | TCGA-DD-AADM-01A | PrimaryTumor | 12 | NA |
| 86 | TCGA-LIHC | TCGA-DD-AADB | TCGA-DD-AADB-01A | PrimaryTumor | NA | 1242 |
| 87 | TCGA-LIHC | TCGA-BD-A3ER | TCGA-BD-A3ER-01A | PrimaryTumor | NA | 1115 |
| 88 | TCGA-LIHC | TCGA-FV-A23B | TCGA-FV-A23B-11A | SolidTissueNormal | 1852 | 987 |
| 89 | TCGA-LIHC | TCGA-DD-A1EL | TCGA-DD-A1EL-11A | SolidTissueNormal | 415 | NA |
| 90 | TCGA-LIHC | TCGA-G3-AAV2 | TCGA-G3-AAV2-01A | PrimaryTumor | NA | 372 |
| 91 | TCGA-LIHC | TCGA-BW-A5NQ | TCGA-BW-A5NQ-01A | PrimaryTumor | NA | NA |
| 92 | TCGA-LIHC | TCGA-BC-A216 | TCGA-BC-A216-11A | SolidTissueNormal | NA | 1351 |
| 93 | TCGA-LIHC | TCGA-DD-AAVQ | TCGA-DD-AAVQ-01A | PrimaryTumor | NA | 2728 |
| 94 | TCGA-LIHC | TCGA-2Y-A9GV | TCGA-2Y-A9GV-01A | PrimaryTumor | 2532 | NA |
| 95 | TCGA-LIHC | TCGA-ED-A7XP | TCGA-ED-A7XP-01A | PrimaryTumor | NA | 400 |
| 96 | TCGA-LIHC | TCGA-DD-AACZ | TCGA-DD-AACZ-01A | PrimaryTumor | 171 | NA |
| 97 | TCGA-LIHC | TCGA-BC-A10R | TCGA-BC-A10R-11A | SolidTissueNormal | 308 | NA |
| 98 | TCGA-LIHC | TCGA-2Y-A9GS | TCGA-2Y-A9GS-01A | PrimaryTumor | 724 | NA |
| 99 | TCGA-LIHC | TCGA-UB-AA0V | TCGA-UB-AA0V-01A | PrimaryTumor | NA | 314 |
| 100 | TCGA-LIHC | TCGA-XR-A8TF | TCGA-XR-A8TF-01A | PrimaryTumor | 693 | NA |
| 101 | TCGA-LIHC | TCGA-DD-AAW2 | TCGA-DD-AAW2-01A | PrimaryTumor | NA | 1855 |
| 102 | TCGA-LIHC | TCGA-2Y-A9H8 | TCGA-2Y-A9H8-01A | PrimaryTumor | 633 | NA |
| 103 | TCGA-LIHC | TCGA-DD-AADF | TCGA-DD-AADF-01A | PrimaryTumor | 115 | NA |
| 104 | TCGA-LIHC | TCGA-DD-AACB | TCGA-DD-AACB-01A | PrimaryTumor | NA | 2324 |
| 105 | TCGA-LIHC | TCGA-WQ-AB4B | TCGA-WQ-AB4B-01A | PrimaryTumor | NA | 395 |
| 106 | TCGA-LIHC | TCGA-HP-A5N0 | TCGA-HP-A5N0-01A | PrimaryTumor | NA | NA |
| 107 | TCGA-LIHC | TCGA-FV-A496 | TCGA-FV-A496-01A | PrimaryTumor | NA | 10 |
| 108 | TCGA-LIHC | TCGA-G3-AAV1 | TCGA-G3-AAV1-01A | PrimaryTumor | 359 | NA |
| 109 | TCGA-LIHC | TCGA-DD-AADN | TCGA-DD-AADN-01A | PrimaryTumor | NA | 898 |
| 110 | TCGA-LIHC | TCGA-FV-A3I0 | TCGA-FV-A3I0-11A | SolidTissueNormal | NA | NA |
| 111 | TCGA-LIHC | TCGA-FV-A4ZQ | TCGA-FV-A4ZQ-01A | PrimaryTumor | NA | 12 |
| 112 | TCGA-LIHC | TCGA-DD-A4NB | TCGA-DD-A4NB-01A | PrimaryTumor | NA | NA |
| 113 | TCGA-LIHC | TCGA-CC-A8HU | TCGA-CC-A8HU-01A | PrimaryTumor | 344 | NA |
| 114 | TCGA-LIHC | TCGA-DD-A11B | TCGA-DD-A11B-11A | SolidTissueNormal | 14 | NA |
| 115 | TCGA-LIHC | TCGA-CC-A3MC | TCGA-CC-A3MC-01A | PrimaryTumor | NA | 363 |
| 116 | TCGA-LIHC | TCGA-DD-AAVY | TCGA-DD-AAVY-01A | PrimaryTumor | NA | 1970 |
| 117 | TCGA-LIHC | TCGA-MI-A75G | TCGA-MI-A75G-01A | PrimaryTumor | NA | 698 |
| 118 | TCGA-LIHC | TCGA-DD-AAE3 | TCGA-DD-AAE3-01A | PrimaryTumor | NA | 566 |
| 119 | TCGA-LIHC | TCGA-DD-AAEH | TCGA-DD-AAEH-01A | PrimaryTumor | NA | 784 |
| 120 | TCGA-LIHC | TCGA-G3-A5SJ | TCGA-G3-A5SJ-01A | PrimaryTumor | NA | 698 |
| 121 | TCGA-LIHC | TCGA-KR-A7K0 | TCGA-KR-A7K0-01A | PrimaryTumor | 65 | NA |
| 122 | TCGA-LIHC | TCGA-DD-A1EH | TCGA-DD-A1EH-11A | SolidTissueNormal | NA | 1495 |
| 123 | TCGA-LIHC | TCGA-BC-A69H | TCGA-BC-A69H-01A | PrimaryTumor | NA | 444 |
| 124 | TCGA-LIHC | TCGA-DD-AACY | TCGA-DD-AACY-01A | PrimaryTumor | NA | 1450 |
| 125 | TCGA-LIHC | TCGA-DD-AAVZ | TCGA-DD-AAVZ-01A | PrimaryTumor | NA | 1900 |
| 126 | TCGA-LIHC | TCGA-DD-A4NL | TCGA-DD-A4NL-01A | PrimaryTumor | NA | 1711 |
| 127 | TCGA-LIHC | TCGA-DD-AADG | TCGA-DD-AADG-01A | PrimaryTumor | NA | 1145 |
| 128 | TCGA-LIHC | TCGA-DD-AAD8 | TCGA-DD-AAD8-01A | PrimaryTumor | NA | 1219 |
| 129 | TCGA-LIHC | TCGA-DD-A4NI | TCGA-DD-A4NI-01A | PrimaryTumor | NA | 816 |
| 130 | TCGA-LIHC | TCGA-CC-A7IK | TCGA-CC-A7IK-01A | PrimaryTumor | 262 | NA |
| 131 | TCGA-LIHC | TCGA-EP-A26S | TCGA-EP-A26S-01A | PrimaryTumor | NA | 608 |
| 132 | TCGA-LIHC | TCGA-DD-AACM | TCGA-DD-AACM-01A | PrimaryTumor | NA | NA |
| 133 | TCGA-LIHC | TCGA-3K-AAZ8 | TCGA-3K-AAZ8-01A | PrimaryTumor | NA | 396 |
| 134 | TCGA-LIHC | TCGA-DD-A1EG | TCGA-DD-A1EG-11A | SolidTissueNormal | 1372 | NA |
| 135 | TCGA-LIHC | TCGA-DD-A1EI | TCGA-DD-A1EI-01A | PrimaryTumor | NA | 183 |
| 136 | TCGA-LIHC | TCGA-DD-A3A9 | TCGA-DD-A3A9-01A | PrimaryTumor | 931 | NA |
| 137 | TCGA-LIHC | TCGA-G3-A25Z | TCGA-G3-A25Z-01A | PrimaryTumor | NA | 655 |
| 138 | TCGA-LIHC | TCGA-5R-AAAM | TCGA-5R-AAAM-01A | PrimaryTumor | 46 | NA |
| 139 | TCGA-LIHC | TCGA-DD-AAC9 | TCGA-DD-AAC9-01A | PrimaryTumor | NA | 347 |
| 140 | TCGA-LIHC | TCGA-DD-A4NQ | TCGA-DD-A4NQ-01A | PrimaryTumor | 373 | NA |
| 141 | TCGA-LIHC | TCGA-EP-A2KB | TCGA-EP-A2KB-01A | PrimaryTumor | 596 | 334 |
| 142 | TCGA-LIHC | TCGA-DD-A113 | TCGA-DD-A113-11A | SolidTissueNormal | NA | 2425 |
| 143 | TCGA-LIHC | TCGA-BC-A216 | TCGA-BC-A216-01A | PrimaryTumor | NA | 1351 |
| 144 | TCGA-LIHC | TCGA-DD-A39W | TCGA-DD-A39W-01A | PrimaryTumor | 827 | NA |
| 145 | TCGA-LIHC | TCGA-XR-A8TE | TCGA-XR-A8TE-01A | PrimaryTumor | NA | 925 |
| 146 | TCGA-LIHC | TCGA-DD-AACD | TCGA-DD-AACD-01A | PrimaryTumor | 381 | NA |
| 147 | TCGA-LIHC | TCGA-DD-AAVU | TCGA-DD-AAVU-01A | PrimaryTumor | NA | 2202 |
| 148 | TCGA-LIHC | TCGA-K7-A5RG | TCGA-K7-A5RG-01A | PrimaryTumor | NA | 519 |
| 149 | TCGA-LIHC | TCGA-DD-AAC8 | TCGA-DD-AAC8-01A | PrimaryTumor | 16 | NA |
| 150 | TCGA-LIHC | TCGA-GJ-A6C0 | TCGA-GJ-A6C0-01A | PrimaryTumor | 31 | NA |
| 151 | TCGA-LIHC | TCGA-CC-A7IG | TCGA-CC-A7IG-01A | PrimaryTumor | 299 | NA |
| 152 | TCGA-LIHC | TCGA-DD-AACW | TCGA-DD-AACW-01A | PrimaryTumor | NA | 1424 |
| 153 | TCGA-LIHC | TCGA-DD-AACG | TCGA-DD-AACG-01A | PrimaryTumor | 469 | NA |
| 154 | TCGA-LIHC | TCGA-DD-A4NO | TCGA-DD-A4NO-01A | PrimaryTumor | NA | 2245 |
| 155 | TCGA-LIHC | TCGA-BD-A2L6 | TCGA-BD-A2L6-11A | SolidTissueNormal | NA | 1363 |
| 156 | TCGA-LIHC | TCGA-DD-A3A5 | TCGA-DD-A3A5-11A | SolidTissueNormal | 3125 | NA |
| 157 | TCGA-LIHC | TCGA-DD-AACI | TCGA-DD-AACI-01A | PrimaryTumor | NA | 1618 |
| 158 | TCGA-LIHC | TCGA-DD-A1ED | TCGA-DD-A1ED-01A | PrimaryTumor | NA | 2301 |
| 159 | TCGA-LIHC | TCGA-2Y-A9GY | TCGA-2Y-A9GY-01A | PrimaryTumor | 757 | NA |
| 160 | TCGA-LIHC | TCGA-G3-AAV3 | TCGA-G3-AAV3-01A | PrimaryTumor | NA | 412 |
| 161 | TCGA-LIHC | TCGA-RC-A7SB | TCGA-RC-A7SB-01A | PrimaryTumor | NA | 588 |
| 162 | TCGA-LIHC | TCGA-DD-AAD2 | TCGA-DD-AAD2-01A | PrimaryTumor | NA | 658 |
| 163 | TCGA-LIHC | TCGA-4R-AA8I | TCGA-4R-AA8I-01A | PrimaryTumor | 262 | 216 |
| 164 | TCGA-LIHC | TCGA-DD-A39X | TCGA-DD-A39X-01A | PrimaryTumor | 1694 | NA |
| 165 | TCGA-LIHC | TCGA-G3-AAUZ | TCGA-G3-AAUZ-01A | PrimaryTumor | NA | 480 |
| 166 | TCGA-LIHC | TCGA-RC-A6M6 | TCGA-RC-A6M6-01A | PrimaryTumor | NA | 9 |
| 167 | TCGA-LIHC | TCGA-RC-A7SK | TCGA-RC-A7SK-01A | PrimaryTumor | NA | 472 |
| 168 | TCGA-LIHC | TCGA-BC-A10W | TCGA-BC-A10W-11A | SolidTissueNormal | 91 | NA |
| 169 | TCGA-LIHC | TCGA-BC-A112 | TCGA-BC-A112-01A | PrimaryTumor | 153 | NA |
| 170 | TCGA-LIHC | TCGA-G3-A25T | TCGA-G3-A25T-01A | PrimaryTumor | NA | 1553 |
| 171 | TCGA-LIHC | TCGA-KR-A7K7 | TCGA-KR-A7K7-01A | PrimaryTumor | NA | 951 |
| 172 | TCGA-LIHC | TCGA-2Y-A9H9 | TCGA-2Y-A9H9-01A | PrimaryTumor | NA | 697 |
| 173 | TCGA-LIHC | TCGA-DD-A1EJ | TCGA-DD-A1EJ-01A | PrimaryTumor | 1005 | 887 |
| 174 | TCGA-LIHC | TCGA-DD-AACJ | TCGA-DD-AACJ-01A | PrimaryTumor | NA | 2102 |
| 175 | TCGA-LIHC | TCGA-G3-AAV6 | TCGA-G3-AAV6-01A | PrimaryTumor | 65 | NA |
| 176 | TCGA-LIHC | TCGA-WJ-A86L | TCGA-WJ-A86L-01A | PrimaryTumor | NA | NA |
| 177 | TCGA-LIHC | TCGA-G3-A3CG | TCGA-G3-A3CG-01A | PrimaryTumor | NA | 673 |
| 178 | TCGA-LIHC | TCGA-UB-A7MA | TCGA-UB-A7MA-01A | PrimaryTumor | NA | NA |
| 179 | TCGA-LIHC | TCGA-O8-A75V | TCGA-O8-A75V-01A | PrimaryTumor | NA | 538 |
| 180 | TCGA-LIHC | TCGA-K7-A6G5 | TCGA-K7-A6G5-01A | PrimaryTumor | NA | 512 |
| 181 | TCGA-LIHC | TCGA-CC-5260 | TCGA-CC-5260-01A | PrimaryTumor | 87 | NA |
| 182 | TCGA-LIHC | TCGA-DD-AADO | TCGA-DD-AADO-01A | PrimaryTumor | NA | 453 |
| 183 | TCGA-LIHC | TCGA-WX-AA44 | TCGA-WX-AA44-01A | PrimaryTumor | NA | 615 |
| 184 | TCGA-LIHC | TCGA-DD-A3A8 | TCGA-DD-A3A8-11A | SolidTissueNormal | 11 | NA |
| 185 | TCGA-LIHC | TCGA-FV-A2QR | TCGA-FV-A2QR-11A | SolidTissueNormal | 581 | NA |
| 186 | TCGA-LIHC | TCGA-5C-AAPD | TCGA-5C-AAPD-01A | PrimaryTumor | NA | NA |
| 187 | TCGA-LIHC | TCGA-BC-A10U | TCGA-BC-A10U-11A | SolidTissueNormal | 837 | NA |
| 188 | TCGA-LIHC | TCGA-CC-A1HT | TCGA-CC-A1HT-01A | PrimaryTumor | 101 | NA |
| 189 | TCGA-LIHC | TCGA-G3-A5SI | TCGA-G3-A5SI-01A | PrimaryTumor | 768 | 447 |
| 190 | TCGA-LIHC | TCGA-DD-AAVV | TCGA-DD-AAVV-01A | PrimaryTumor | NA | 2455 |
| 191 | TCGA-LIHC | TCGA-DD-AAEA | TCGA-DD-AAEA-01A | PrimaryTumor | NA | 575 |
| 192 | TCGA-LIHC | TCGA-DD-AACE | TCGA-DD-AACE-01A | PrimaryTumor | NA | 2184 |
| 193 | TCGA-LIHC | TCGA-BD-A2L6 | TCGA-BD-A2L6-01A | PrimaryTumor | NA | 1363 |
| 194 | TCGA-LIHC | TCGA-DD-AAD6 | TCGA-DD-AAD6-01A | PrimaryTumor | NA | 672 |
| 195 | TCGA-LIHC | TCGA-CC-A9FS | TCGA-CC-A9FS-01A | PrimaryTumor | NA | 211 |
| 196 | TCGA-LIHC | TCGA-DD-AAD0 | TCGA-DD-AAD0-01A | PrimaryTumor | NA | 137 |
| 197 | TCGA-LIHC | TCGA-2Y-A9H2 | TCGA-2Y-A9H2-01A | PrimaryTumor | NA | 1731 |
| 198 | TCGA-LIHC | TCGA-DD-AAEK | TCGA-DD-AAEK-01A | PrimaryTumor | NA | 1067 |
| 199 | TCGA-LIHC | TCGA-BC-A10U | TCGA-BC-A10U-01A | PrimaryTumor | 837 | NA |
| 200 | TCGA-LIHC | TCGA-ZP-A9D0 | TCGA-ZP-A9D0-01A | PrimaryTumor | NA | 1091 |
| 201 | TCGA-LIHC | TCGA-CC-A8HT | TCGA-CC-A8HT-01A | PrimaryTumor | 140 | NA |
| 202 | TCGA-LIHC | TCGA-EP-A3RK | TCGA-EP-A3RK-01A | PrimaryTumor | NA | 363 |
| 203 | TCGA-LIHC | TCGA-G3-A7M5 | TCGA-G3-A7M5-01A | PrimaryTumor | NA | 447 |
| 204 | TCGA-LIHC | TCGA-MI-A75H | TCGA-MI-A75H-01A | PrimaryTumor | NA | 747 |
| 205 | TCGA-LIHC | TCGA-DD-A1EI | TCGA-DD-A1EI-11A | SolidTissueNormal | NA | 183 |
| 206 | TCGA-LIHC | TCGA-LG-A6GG | TCGA-LG-A6GG-01A | PrimaryTumor | NA | 387 |
| 207 | TCGA-LIHC | TCGA-CC-A7IF | TCGA-CC-A7IF-01A | PrimaryTumor | 649 | NA |
| 208 | TCGA-LIHC | TCGA-ZP-A9CZ | TCGA-ZP-A9CZ-01A | PrimaryTumor | NA | 706 |
| 209 | TCGA-LIHC | TCGA-XR-A8TG | TCGA-XR-A8TG-01A | PrimaryTumor | NA | 898 |
| 210 | TCGA-LIHC | TCGA-DD-A4NA | TCGA-DD-A4NA-01A | PrimaryTumor | NA | 1008 |
| 211 | TCGA-LIHC | TCGA-ED-A8O6 | TCGA-ED-A8O6-01A | PrimaryTumor | 56 | 6 |
| 212 | TCGA-LIHC | TCGA-DD-A11D | TCGA-DD-A11D-01A | PrimaryTumor | 1560 | NA |
| 213 | TCGA-LIHC | TCGA-G3-A7M7 | TCGA-G3-A7M7-01A | PrimaryTumor | NA | 361 |
| 214 | TCGA-LIHC | TCGA-DD-AACL | TCGA-DD-AACL-01A | PrimaryTumor | 107 | 58 |
| 215 | TCGA-LIHC | TCGA-DD-A39Y | TCGA-DD-A39Y-01A | PrimaryTumor | 171 | NA |
| 216 | TCGA-LIHC | TCGA-DD-AACT | TCGA-DD-AACT-01A | PrimaryTumor | NA | 1562 |
| 217 | TCGA-LIHC | TCGA-FV-A3I1 | TCGA-FV-A3I1-01A | PrimaryTumor | 247 | NA |
| 218 | TCGA-LIHC | TCGA-DD-A4NS | TCGA-DD-A4NS-01A | PrimaryTumor | 2456 | NA |
| 219 | TCGA-LIHC | TCGA-MI-A75E | TCGA-MI-A75E-01A | PrimaryTumor | NA | 507 |
| 220 | TCGA-LIHC | TCGA-CC-A8HV | TCGA-CC-A8HV-01A | PrimaryTumor | 279 | NA |
| 221 | TCGA-LIHC | TCGA-DD-A1EE | TCGA-DD-A1EE-11A | SolidTissueNormal | 349 | 204 |
| 222 | TCGA-LIHC | TCGA-DD-AACQ | TCGA-DD-AACQ-01A | PrimaryTumor | 432 | 429 |
| 223 | TCGA-LIHC | TCGA-BC-A10S | TCGA-BC-A10S-01A | PrimaryTumor | 1423 | NA |
| 224 | TCGA-LIHC | TCGA-DD-A11A | TCGA-DD-A11A-01A | PrimaryTumor | NA | 79 |
| 225 | TCGA-LIHC | TCGA-FV-A3I1 | TCGA-FV-A3I1-11A | SolidTissueNormal | 247 | NA |
| 226 | TCGA-LIHC | TCGA-2Y-A9H4 | TCGA-2Y-A9H4-01A | PrimaryTumor | NA | 1452 |
| 227 | TCGA-LIHC | TCGA-2Y-A9GW | TCGA-2Y-A9GW-01A | PrimaryTumor | 1271 | NA |
| 228 | TCGA-LIHC | TCGA-FV-A3R2 | TCGA-FV-A3R2-11A | SolidTissueNormal | 194 | NA |
| 229 | TCGA-LIHC | TCGA-DD-AACK | TCGA-DD-AACK-01A | PrimaryTumor | NA | 9 |
| 230 | TCGA-LIHC | TCGA-CC-A3MB | TCGA-CC-A3MB-01A | PrimaryTumor | 315 | 3 |
| 231 | TCGA-LIHC | TCGA-G3-A3CK | TCGA-G3-A3CK-01A | PrimaryTumor | NA | 585 |
| 232 | TCGA-LIHC | TCGA-BW-A5NP | TCGA-BW-A5NP-01A | PrimaryTumor | NA | NA |
| 233 | TCGA-LIHC | TCGA-CC-5263 | TCGA-CC-5263-01A | PrimaryTumor | 129 | NA |
| 234 | TCGA-LIHC | TCGA-BD-A3EP | TCGA-BD-A3EP-01A | PrimaryTumor | NA | 409 |
| 235 | TCGA-LIHC | TCGA-DD-A3A2 | TCGA-DD-A3A2-01A | PrimaryTumor | 2131 | NA |
| 236 | TCGA-LIHC | TCGA-DD-A115 | TCGA-DD-A115-01A | PrimaryTumor | 2542 | NA |
| 237 | TCGA-LIHC | TCGA-G3-A3CH | TCGA-G3-A3CH-11A | SolidTissueNormal | NA | 780 |
| 238 | TCGA-LIHC | TCGA-RC-A7SH | TCGA-RC-A7SH-01A | PrimaryTumor | NA | NA |
| 239 | TCGA-LIHC | TCGA-CC-5264 | TCGA-CC-5264-01A | PrimaryTumor | 102 | NA |
| 240 | TCGA-LIHC | TCGA-BC-A3KG | TCGA-BC-A3KG-01A | PrimaryTumor | NA | 680 |
| 241 | TCGA-LIHC | TCGA-CC-A9FW | TCGA-CC-A9FW-01A | PrimaryTumor | NA | 248 |
| 242 | TCGA-LIHC | TCGA-DD-AADI | TCGA-DD-AADI-01A | PrimaryTumor | NA | 1085 |
| 243 | TCGA-LIHC | TCGA-DD-AACS | TCGA-DD-AACS-01A | PrimaryTumor | NA | 1804 |
| 244 | TCGA-LIHC | TCGA-ZP-A9D4 | TCGA-ZP-A9D4-01A | PrimaryTumor | NA | 395 |
| 245 | TCGA-LIHC | TCGA-DD-A1EC | TCGA-DD-A1EC-11A | SolidTissueNormal | NA | 602 |
| 246 | TCGA-LIHC | TCGA-5R-AA1D | TCGA-5R-AA1D-01A | PrimaryTumor | NA | 449 |
| 247 | TCGA-LIHC | TCGA-NI-A4U2 | TCGA-NI-A4U2-01A | PrimaryTumor | 1791 | NA |
| 248 | TCGA-LIHC | TCGA-5R-AA1C | TCGA-5R-AA1C-01A | PrimaryTumor | NA | 520 |
| 249 | TCGA-LIHC | TCGA-BW-A5NO | TCGA-BW-A5NO-01A | PrimaryTumor | NA | 20 |
| 250 | TCGA-LIHC | TCGA-BC-A10T | TCGA-BC-A10T-01A | PrimaryTumor | 837 | NA |
| 251 | TCGA-LIHC | TCGA-5C-A9VH | TCGA-5C-A9VH-01A | PrimaryTumor | NA | 322 |
| 252 | TCGA-LIHC | TCGA-RC-A6M5 | TCGA-RC-A6M5-01A | PrimaryTumor | NA | NA |
| 253 | TCGA-LIHC | TCGA-DD-A73A | TCGA-DD-A73A-01A | PrimaryTumor | NA | 728 |
| 254 | TCGA-LIHC | TCGA-DD-A1EK | TCGA-DD-A1EK-01A | PrimaryTumor | 558 | NA |
| 255 | TCGA-LIHC | TCGA-DD-AADA | TCGA-DD-AADA-01A | PrimaryTumor | NA | 1233 |
| 256 | TCGA-LIHC | TCGA-DD-A3A3 | TCGA-DD-A3A3-11A | SolidTissueNormal | 535 | NA |
| 257 | TCGA-LIHC | TCGA-DD-AAED | TCGA-DD-AAED-01A | PrimaryTumor | NA | 763 |
| 258 | TCGA-LIHC | TCGA-DD-AADW | TCGA-DD-AADW-01A | PrimaryTumor | NA | 587 |
| 259 | TCGA-LIHC | TCGA-BD-A3EP | TCGA-BD-A3EP-11A | SolidTissueNormal | NA | 409 |
| 260 | TCGA-LIHC | TCGA-BC-4072 | TCGA-BC-4072-01B | PrimaryTumor | 1490 | NA |
| 261 | TCGA-LIHC | TCGA-FV-A4ZP | TCGA-FV-A4ZP-01A | PrimaryTumor | 2486 | 1836 |
| 262 | TCGA-LIHC | TCGA-DD-AAE2 | TCGA-DD-AAE2-01A | PrimaryTumor | NA | 638 |
| 263 | TCGA-LIHC | TCGA-DD-A1EB | TCGA-DD-A1EB-11A | SolidTissueNormal | NA | 2017 |
| 264 | TCGA-LIHC | TCGA-ED-A97K | TCGA-ED-A97K-01A | PrimaryTumor | NA | NA |
| 265 | TCGA-LIHC | TCGA-DD-A39Z | TCGA-DD-A39Z-11A | SolidTissueNormal | 601 | NA |
| 266 | TCGA-LIHC | TCGA-2Y-A9H0 | TCGA-2Y-A9H0-01A | PrimaryTumor | NA | 3675 |
| 267 | TCGA-LIHC | TCGA-BC-A217 | TCGA-BC-A217-01A | PrimaryTumor | 1397 | 421 |
| 268 | TCGA-LIHC | TCGA-DD-A4NP | TCGA-DD-A4NP-01A | PrimaryTumor | NA | 3308 |
| 269 | TCGA-LIHC | TCGA-2V-A95S | TCGA-2V-A95S-01A | PrimaryTumor | NA | NA |
| 270 | TCGA-LIHC | TCGA-DD-AADD | TCGA-DD-AADD-01A | PrimaryTumor | NA | 1231 |
| 271 | TCGA-LIHC | TCGA-BC-A8YO | TCGA-BC-A8YO-01A | PrimaryTumor | NA | 562 |
| 272 | TCGA-LIHC | TCGA-DD-AAVR | TCGA-DD-AAVR-01A | PrimaryTumor | NA | 2513 |
| 273 | TCGA-LIHC | TCGA-DD-A11D | TCGA-DD-A11D-11A | SolidTissueNormal | 1560 | NA |
| 274 | TCGA-LIHC | TCGA-UB-A7MB | TCGA-UB-A7MB-01A | PrimaryTumor | NA | 601 |
| 275 | TCGA-LIHC | TCGA-DD-AAVX | TCGA-DD-AAVX-01A | PrimaryTumor | NA | 1718 |
| 276 | TCGA-LIHC | TCGA-CC-A7IE | TCGA-CC-A7IE-01A | PrimaryTumor | 217 | NA |
| 277 | TCGA-LIHC | TCGA-DD-AADK | TCGA-DD-AADK-01A | PrimaryTumor | NA | 1049 |
| 278 | TCGA-LIHC | TCGA-DD-AAVS | TCGA-DD-AAVS-01A | PrimaryTumor | NA | 1823 |
| 279 | TCGA-LIHC | TCGA-DD-A3A3 | TCGA-DD-A3A3-01A | PrimaryTumor | 535 | NA |
| 280 | TCGA-LIHC | TCGA-DD-A11A | TCGA-DD-A11A-11A | SolidTissueNormal | NA | 79 |
| 281 | TCGA-LIHC | TCGA-DD-AACP | TCGA-DD-AACP-01A | PrimaryTumor | NA | 415 |
| 282 | TCGA-LIHC | TCGA-DD-A73G | TCGA-DD-A73G-01A | PrimaryTumor | NA | 3478 |
| 283 | TCGA-LIHC | TCGA-DD-AAEI | TCGA-DD-AAEI-01A | PrimaryTumor | NA | 1531 |
| 284 | TCGA-LIHC | TCGA-2Y-A9H6 | TCGA-2Y-A9H6-01A | PrimaryTumor | NA | 357 |
| 285 | TCGA-LIHC | TCGA-DD-A1EJ | TCGA-DD-A1EJ-11A | SolidTissueNormal | 1005 | 887 |
| 286 | TCGA-LIHC | TCGA-DD-AACN | TCGA-DD-AACN-01A | PrimaryTumor | NA | 1302 |
| 287 | TCGA-LIHC | TCGA-2Y-A9H3 | TCGA-2Y-A9H3-01A | PrimaryTumor | NA | 1516 |
| 288 | TCGA-LIHC | TCGA-LG-A9QD | TCGA-LG-A9QD-01A | PrimaryTumor | NA | 366 |
| 289 | TCGA-LIHC | TCGA-DD-A3A6 | TCGA-DD-A3A6-01A | PrimaryTumor | 3258 | NA |
| 290 | TCGA-LIHC | TCGA-DD-A116 | TCGA-DD-A116-11A | SolidTissueNormal | 1622 | NA |
| 291 | TCGA-LIHC | TCGA-DD-A39V | TCGA-DD-A39V-11A | SolidTissueNormal | 643 | NA |
| 292 | TCGA-LIHC | TCGA-DD-AACV | TCGA-DD-AACV-01A | PrimaryTumor | NA | 1531 |
| 293 | TCGA-LIHC | TCGA-EP-A2KA | TCGA-EP-A2KA-01A | PrimaryTumor | 627 | 357 |
| 294 | TCGA-LIHC | TCGA-ED-A8O5 | TCGA-ED-A8O5-01A | PrimaryTumor | NA | 406 |
| 295 | TCGA-LIHC | TCGA-EP-A3RK | TCGA-EP-A3RK-11A | SolidTissueNormal | NA | 363 |
| 296 | TCGA-LIHC | TCGA-DD-AADC | TCGA-DD-AADC-01A | PrimaryTumor | 425 | NA |
| 297 | TCGA-LIHC | TCGA-DD-AADV | TCGA-DD-AADV-01A | PrimaryTumor | NA | 574 |
| 298 | TCGA-LIHC | TCGA-G3-A25U | TCGA-G3-A25U-01A | PrimaryTumor | NA | 1636 |
| 299 | TCGA-LIHC | TCGA-UB-A7MC | TCGA-UB-A7MC-01A | PrimaryTumor | NA | 500 |
| 300 | TCGA-LIHC | TCGA-ED-A66Y | TCGA-ED-A66Y-01A | PrimaryTumor | 296 | 7 |
| 301 | TCGA-LIHC | TCGA-DD-A119 | TCGA-DD-A119-01A | PrimaryTumor | 223 | NA |
| 302 | TCGA-LIHC | TCGA-DD-A39V | TCGA-DD-A39V-01A | PrimaryTumor | 643 | NA |
| 303 | TCGA-LIHC | TCGA-CC-A7IJ | TCGA-CC-A7IJ-01A | PrimaryTumor | NA | 382 |
| 304 | TCGA-LIHC | TCGA-BC-A10R | TCGA-BC-A10R-01A | PrimaryTumor | 308 | NA |
| 305 | TCGA-LIHC | TCGA-DD-A1EC | TCGA-DD-A1EC-01A | PrimaryTumor | NA | 602 |
| 306 | TCGA-LIHC | TCGA-K7-A5RF | TCGA-K7-A5RF-01A | PrimaryTumor | NA | 631 |
| 307 | TCGA-LIHC | TCGA-DD-A73B | TCGA-DD-A73B-01A | PrimaryTumor | 283 | NA |
| 308 | TCGA-LIHC | TCGA-ES-A2HT | TCGA-ES-A2HT-01A | PrimaryTumor | 438 | NA |
| 309 | TCGA-LIHC | TCGA-ES-A2HT | TCGA-ES-A2HT-11A | SolidTissueNormal | 438 | NA |
| 310 | TCGA-LIHC | TCGA-DD-AADE | TCGA-DD-AADE-01A | PrimaryTumor | NA | NA |
| 311 | TCGA-LIHC | TCGA-EP-A12J | TCGA-EP-A12J-01A | PrimaryTumor | NA | 570 |
| 312 | TCGA-LIHC | TCGA-UB-A7ME | TCGA-UB-A7ME-01A | PrimaryTumor | NA | 486 |
| 313 | TCGA-LIHC | TCGA-DD-AACX | TCGA-DD-AACX-01A | PrimaryTumor | NA | 170 |
| 314 | TCGA-LIHC | TCGA-WQ-A9G7 | TCGA-WQ-A9G7-01A | PrimaryTumor | NA | 30 |
| 315 | TCGA-LIHC | TCGA-CC-5261 | TCGA-CC-5261-01A | PrimaryTumor | 97 | 12 |
| 316 | TCGA-LIHC | TCGA-FV-A495 | TCGA-FV-A495-01A | PrimaryTumor | NA | 1 |
| 317 | TCGA-LIHC | TCGA-DD-A3A6 | TCGA-DD-A3A6-11A | SolidTissueNormal | 3258 | NA |
| 318 | TCGA-LIHC | TCGA-CC-A7II | TCGA-CC-A7II-01A | PrimaryTumor | NA | 399 |
| 319 | TCGA-LIHC | TCGA-CC-A5UE | TCGA-CC-A5UE-01A | PrimaryTumor | 272 | NA |
| 320 | TCGA-LIHC | TCGA-DD-AAEE | TCGA-DD-AAEE-01A | PrimaryTumor | NA | 810 |
| 321 | TCGA-LIHC | TCGA-G3-A25Y | TCGA-G3-A25Y-01A | PrimaryTumor | 452 | NA |
| 322 | TCGA-LIHC | TCGA-BC-A5W4 | TCGA-BC-A5W4-01A | PrimaryTumor | 547 | 289 |
| 323 | TCGA-LIHC | TCGA-DD-A4NV | TCGA-DD-A4NV-01A | PrimaryTumor | NA | 2398 |
| 324 | TCGA-LIHC | TCGA-DD-AAD5 | TCGA-DD-AAD5-01A | PrimaryTumor | NA | 1345 |
| 325 | TCGA-LIHC | TCGA-CC-A7IL | TCGA-CC-A7IL-01A | PrimaryTumor | 278 | NA |
| 326 | TCGA-LIHC | TCGA-G3-A25V | TCGA-G3-A25V-01A | PrimaryTumor | NA | 860 |
| 327 | TCGA-LIHC | TCGA-G3-A6UC | TCGA-G3-A6UC-01A | PrimaryTumor | NA | 671 |
| 328 | TCGA-LIHC | TCGA-DD-A39W | TCGA-DD-A39W-11A | SolidTissueNormal | 827 | NA |
| 329 | TCGA-LIHC | TCGA-XR-A8TD | TCGA-XR-A8TD-01A | PrimaryTumor | NA | NA |
| 330 | TCGA-LIHC | TCGA-BC-A10X | TCGA-BC-A10X-01A | PrimaryTumor | 770 | NA |
| 331 | TCGA-LIHC | TCGA-G3-A7M6 | TCGA-G3-A7M6-01A | PrimaryTumor | NA | 632 |
| 332 | TCGA-LIHC | TCGA-DD-AAE0 | TCGA-DD-AAE0-01A | PrimaryTumor | NA | 555 |
| 333 | TCGA-LIHC | TCGA-G3-A5SK | TCGA-G3-A5SK-01A | PrimaryTumor | NA | 744 |
| 334 | TCGA-LIHC | TCGA-MI-A75I | TCGA-MI-A75I-01A | PrimaryTumor | NA | 630 |
| 335 | TCGA-LIHC | TCGA-FV-A3I0 | TCGA-FV-A3I0-01A | PrimaryTumor | NA | NA |
| 336 | TCGA-LIHC | TCGA-DD-A4NH | TCGA-DD-A4NH-01A | PrimaryTumor | NA | 917 |
| 337 | TCGA-LIHC | TCGA-BC-A10T | TCGA-BC-A10T-11A | SolidTissueNormal | 837 | NA |
| 338 | TCGA-LIHC | TCGA-DD-AACC | TCGA-DD-AACC-01A | PrimaryTumor | 1685 | NA |
| 339 | TCGA-LIHC | TCGA-DD-A3A5 | TCGA-DD-A3A5-01A | PrimaryTumor | 3125 | NA |
| 340 | TCGA-LIHC | TCGA-DD-A11B | TCGA-DD-A11B-01A | PrimaryTumor | 14 | NA |
| 341 | TCGA-LIHC | TCGA-DD-AAE6 | TCGA-DD-AAE6-01A | PrimaryTumor | NA | 141 |
| 342 | TCGA-LIHC | TCGA-DD-A39Z | TCGA-DD-A39Z-01A | PrimaryTumor | 601 | NA |
| 343 | TCGA-LIHC | TCGA-CC-A9FU | TCGA-CC-A9FU-01A | PrimaryTumor | NA | NA |
| 344 | TCGA-LIHC | TCGA-GJ-A9DB | TCGA-GJ-A9DB-01A | PrimaryTumor | 67 | NA |
| 345 | TCGA-LIHC | TCGA-DD-AAW3 | TCGA-DD-AAW3-01A | PrimaryTumor | NA | 1633 |
| 346 | TCGA-LIHC | TCGA-ZP-A9CV | TCGA-ZP-A9CV-01A | PrimaryTumor | 1088 | 142 |
| 347 | TCGA-LIHC | TCGA-DD-A1EG | TCGA-DD-A1EG-01A | PrimaryTumor | 1372 | NA |
| 348 | TCGA-LIHC | TCGA-DD-AADP | TCGA-DD-AADP-01A | PrimaryTumor | NA | 458 |
| 349 | TCGA-LIHC | TCGA-G3-A3CH | TCGA-G3-A3CH-01A | PrimaryTumor | NA | 780 |
| 350 | TCGA-LIHC | TCGA-G3-A7M9 | TCGA-G3-A7M9-01A | PrimaryTumor | 56 | NA |
| 351 | TCGA-LIHC | TCGA-ED-A7PY | TCGA-ED-A7PY-01A | PrimaryTumor | NA | 390 |
| 352 | TCGA-LIHC | TCGA-DD-AAD1 | TCGA-DD-AAD1-01A | PrimaryTumor | NA | 564 |
| 353 | TCGA-LIHC | TCGA-UB-A7MF | TCGA-UB-A7MF-01A | PrimaryTumor | 214 | NA |
| 354 | TCGA-LIHC | TCGA-2Y-A9HB | TCGA-2Y-A9HB-01A | PrimaryTumor | NA | 260 |
| 355 | TCGA-LIHC | TCGA-DD-A1EL | TCGA-DD-A1EL-01A | PrimaryTumor | 415 | NA |
| 356 | TCGA-LIHC | TCGA-NI-A8LF | TCGA-NI-A8LF-01A | PrimaryTumor | NA | 799 |
| 357 | TCGA-LIHC | TCGA-DD-AAVW | TCGA-DD-AAVW-01A | PrimaryTumor | NA | 2317 |
| 358 | TCGA-LIHC | TCGA-CC-A7IH | TCGA-CC-A7IH-01A | PrimaryTumor | NA | 365 |
| 359 | TCGA-LIHC | TCGA-DD-A1EA | TCGA-DD-A1EA-01A | PrimaryTumor | NA | 2415 |
| 360 | TCGA-LIHC | TCGA-ED-A459 | TCGA-ED-A459-01A | PrimaryTumor | NA | 910 |
| 361 | TCGA-LIHC | TCGA-RC-A6M4 | TCGA-RC-A6M4-01A | PrimaryTumor | NA | 22 |
| 362 | TCGA-LIHC | TCGA-ED-A627 | TCGA-ED-A627-01A | PrimaryTumor | NA | 423 |
| 363 | TCGA-LIHC | TCGA-DD-AAEB | TCGA-DD-AAEB-01A | PrimaryTumor | NA | 478 |
| 364 | TCGA-LIHC | TCGA-DD-AADQ | TCGA-DD-AADQ-01A | PrimaryTumor | NA | 436 |
| 365 | TCGA-LIHC | TCGA-2Y-A9H1 | TCGA-2Y-A9H1-01A | PrimaryTumor | 1229 | NA |
| 366 | TCGA-LIHC | TCGA-CC-5262 | TCGA-CC-5262-01A | PrimaryTumor | 103 | NA |
| 367 | TCGA-LIHC | TCGA-RC-A7SF | TCGA-RC-A7SF-01A | PrimaryTumor | NA | 579 |
| 368 | TCGA-LIHC | TCGA-DD-A113 | TCGA-DD-A113-01A | PrimaryTumor | NA | 2425 |
| 369 | TCGA-LIHC | TCGA-DD-AA3A | TCGA-DD-AA3A-01A | PrimaryTumor | NA | NA |
| 370 | TCGA-LIHC | TCGA-CC-A5UC | TCGA-CC-A5UC-01A | PrimaryTumor | 347 | NA |
| 371 | TCGA-LIHC | TCGA-G3-A25S | TCGA-G3-A25S-01A | PrimaryTumor | 416 | 37 |
| 372 | TCGA-LIHC | TCGA-DD-A4NR | TCGA-DD-A4NR-01A | PrimaryTumor | 9 | NA |
| 373 | TCGA-LIHC | TCGA-CC-A9FV | TCGA-CC-A9FV-01A | PrimaryTumor | NA | NA |
| 374 | TCGA-LIHC | TCGA-DD-AADU | TCGA-DD-AADU-01A | PrimaryTumor | NA | 554 |
| 375 | TCGA-LIHC | TCGA-G3-A3CI | TCGA-G3-A3CI-01A | PrimaryTumor | NA | 180 |
| 376 | TCGA-LIHC | TCGA-CC-A3M9 | TCGA-CC-A3M9-01A | PrimaryTumor | NA | NA |
| 377 | TCGA-LIHC | TCGA-DD-AACA | TCGA-DD-AACA-01A | PrimaryTumor | NA | 2301 |
| 378 | TCGA-LIHC | TCGA-EP-A26S | TCGA-EP-A26S-11A | SolidTissueNormal | NA | 608 |
| 379 | TCGA-LIHC | TCGA-ED-A82E | TCGA-ED-A82E-01A | PrimaryTumor | NA | 408 |
| 380 | TCGA-LIHC | TCGA-BC-A10Z | TCGA-BC-A10Z-01A | PrimaryTumor | 34 | NA |
| 381 | TCGA-LIHC | TCGA-DD-A11C | TCGA-DD-A11C-01A | PrimaryTumor | NA | 662 |
| 382 | TCGA-LIHC | TCGA-ZP-A9D1 | TCGA-ZP-A9D1-01A | PrimaryTumor | NA | 21 |
| 383 | TCGA-LIHC | TCGA-2Y-A9HA | TCGA-2Y-A9HA-01A | PrimaryTumor | 36 | NA |
| 384 | TCGA-LIHC | TCGA-ED-A66X | TCGA-ED-A66X-01A | PrimaryTumor | NA | 406 |
| 385 | TCGA-LIHC | TCGA-QA-A7B7 | TCGA-QA-A7B7-01A | PrimaryTumor | NA | 94 |
| 386 | TCGA-LIHC | TCGA-WX-AA46 | TCGA-WX-AA46-01A | PrimaryTumor | NA | 756 |
| 387 | TCGA-LIHC | TCGA-DD-AAE1 | TCGA-DD-AAE1-01A | PrimaryTumor | NA | 552 |
| 388 | TCGA-LIHC | TCGA-ES-A2HS | TCGA-ES-A2HS-01A | PrimaryTumor | 688 | NA |
| 389 | TCGA-LIHC | TCGA-DD-A3A0 | TCGA-DD-A3A0-01A | PrimaryTumor | NA | NA |
| 390 | TCGA-LIHC | TCGA-DD-A118 | TCGA-DD-A118-11A | SolidTissueNormal | NA | 3437 |
| 391 | TCGA-LIHC | TCGA-HP-A5MZ | TCGA-HP-A5MZ-01A | PrimaryTumor | 91 | NA |
| 392 | TCGA-LIHC | TCGA-DD-AACH | TCGA-DD-AACH-01A | PrimaryTumor | 195 | NA |
| 393 | TCGA-LIHC | TCGA-G3-A25W | TCGA-G3-A25W-01A | PrimaryTumor | NA | NA |
| 394 | TCGA-LIHC | TCGA-DD-A3A8 | TCGA-DD-A3A8-01A | PrimaryTumor | 11 | NA |
| 395 | TCGA-LIHC | TCGA-G3-AAV0 | TCGA-G3-AAV0-01A | PrimaryTumor | NA | 476 |
| 396 | TCGA-LIHC | TCGA-KR-A7K2 | TCGA-KR-A7K2-01A | PrimaryTumor | NA | NA |
| 397 | TCGA-LIHC | TCGA-DD-A73F | TCGA-DD-A73F-01A | PrimaryTumor | NA | 1085 |
| 398 | TCGA-LIHC | TCGA-DD-AACF | TCGA-DD-AACF-01A | PrimaryTumor | 365 | NA |
| 399 | TCGA-LIHC | TCGA-DD-AAVP | TCGA-DD-AAVP-01A | PrimaryTumor | NA | 2752 |
| 400 | TCGA-LIHC | TCGA-KR-A7K8 | TCGA-KR-A7K8-01A | PrimaryTumor | NA | 906 |
| 401 | TCGA-LIHC | TCGA-2Y-A9H7 | TCGA-2Y-A9H7-01A | PrimaryTumor | NA | 1168 |
| 402 | TCGA-LIHC | TCGA-ZS-A9CF | TCGA-ZS-A9CF-01A | PrimaryTumor | NA | 2412 |
| 403 | TCGA-LIHC | TCGA-CC-A123 | TCGA-CC-A123-01A | PrimaryTumor | NA | 219 |
| 404 | TCGA-LIHC | TCGA-BC-A10Q | TCGA-BC-A10Q-01A | PrimaryTumor | 1135 | NA |
| 405 | TCGA-LIHC | TCGA-ED-A5KG | TCGA-ED-A5KG-01A | PrimaryTumor | NA | 854 |
| 406 | TCGA-LIHC | TCGA-BC-A10Z | TCGA-BC-A10Z-11A | SolidTissueNormal | 34 | NA |
| 407 | TCGA-LIHC | TCGA-DD-A116 | TCGA-DD-A116-01A | PrimaryTumor | 1622 | NA |
| 408 | TCGA-LIHC | TCGA-2Y-A9GT | TCGA-2Y-A9GT-01A | PrimaryTumor | 1624 | NA |
| 409 | TCGA-LIHC | TCGA-G3-A7M8 | TCGA-G3-A7M8-01A | PrimaryTumor | NA | 430 |
| 410 | TCGA-LIHC | TCGA-BC-A10Y | TCGA-BC-A10Y-11A | SolidTissueNormal | 711 | NA |
| 411 | TCGA-LIHC | TCGA-DD-A3A2 | TCGA-DD-A3A2-11A | SolidTissueNormal | 2131 | NA |
| 412 | TCGA-LIHC | TCGA-DD-A114 | TCGA-DD-A114-11A | SolidTissueNormal | 1149 | NA |
| 413 | TCGA-LIHC | TCGA-K7-AAU7 | TCGA-K7-AAU7-01A | PrimaryTumor | NA | NA |
| 414 | TCGA-LIHC | TCGA-MR-A520 | TCGA-MR-A520-01A | PrimaryTumor | NA | 229 |
| 415 | TCGA-LIHC | TCGA-G3-A5SL | TCGA-G3-A5SL-01A | PrimaryTumor | NA | 621 |
| 416 | TCGA-LIHC | TCGA-ED-A7XO | TCGA-ED-A7XO-01A | PrimaryTumor | NA | 427 |
| 417 | TCGA-LIHC | TCGA-DD-AADR | TCGA-DD-AADR-01A | PrimaryTumor | NA | 2028 |
| 418 | TCGA-LIHC | TCGA-ZS-A9CG | TCGA-ZS-A9CG-01A | PrimaryTumor | NA | 341 |
| 419 | TCGA-LIHC | TCGA-MR-A8JO | TCGA-MR-A8JO-01A | PrimaryTumor | NA | NA |
| 420 | TCGA-LIHC | TCGA-DD-A1EF | TCGA-DD-A1EF-01A | PrimaryTumor | 394 | NA |
| 421 | TCGA-LIHC | TCGA-G3-AAV7 | TCGA-G3-AAV7-01A | PrimaryTumor | NA | 361 |
| 422 | TCGA-LIHC | TCGA-DD-AADS | TCGA-DD-AADS-01A | PrimaryTumor | NA | 474 |
